# On the Cause and Consequences of Coinfection: A General Mechanistic Framework of Within‐Host Parasite Competition

**DOI:** 10.1111/ele.70104

**Published:** 2025-06-27

**Authors:** Ashwini Ramesh, Spencer R. Hall

**Affiliations:** ^1^ Department of Biology Indiana University Bloomington Indiana USA; ^2^ Department of Integrative Biology Michigan State University East Lansing Michigan USA

**Keywords:** coinfection, competition, founder control, host health, niche models, nullclines, nutrient supply, nutritional immunity, priority effects

## Abstract

Coinfections pose serious threats to health and exacerbate parasite burden. If coinfection is detrimental, then what within‐host factors facilitate it? Equally importantly, what hinders it, say via exclusion or priority effects? Such interactions ought to stem from their within‐host environment (‘niche’), that is, resources that parasites steal from hosts and immune cells that kill them. Yet, despite two decades of empirical focus on within‐host infection dynamics, we lack a mechanistic framework to understand why coinfection arises and the diverse range of its' consequences. By applying ecological niche theory, our within‐host competition models lay the foundational theoretical framework for pathogen coinfection. Here, we outline various within‐host competition models. Then, we emphasise general principles arising from an example of a trait‐based niche model of joint resource and apparent competition for immune cells. In this model, coinfection requires a competition‐resistance trade‐off, that each parasite most impacts the factor to which its fitness is most sensitive, and intermediate resource supply. These predictions then provide mechanistic interpretations for questions about the outcomes of various experiments: Why does nutrient supplementation shift relative frequencies of coinfecting parasites? When and how does the sequence of parasite invasion allow only early invading parasites to win? How does intrinsic variation in immune response shape coinfection burden? Together, this mechanistic framework of parasite competition offers new perspectives to better predict within‐host coinfection dynamics through an ecological lens.

## Graphical abstract

By applying ecological niche theory, our work illustrates the foundational principles for understanding pathogen coinfection via the lens of resource and immune (apparent) competition. Coinfection, that is, within‐host species coexistence, necessitates a competition‐resistance trade‐off and that each parasite primarily affects the niche factor to which it is most sensitive. We then synthesised how those forms of competition underlie a comprehensive within‐host framework that offers explanations for numerous puzzling coinfection experiments from the past three decades.

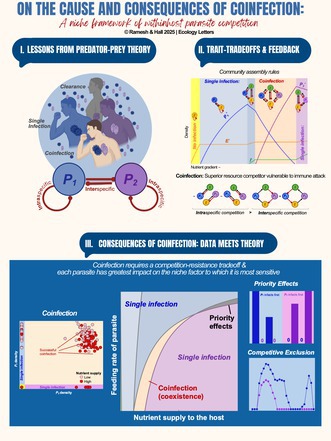

## 1 Introduction

Coinfections pose a serious threat to health for hosts both at the individual and population scale. Coinfection is the concurrent infestation of a host with two or more parasite species. Coinfection worsens human health (76% of 2000 publications in a meta‐analysis) and exacerbates infection burden (57%; Griffiths et al. 2011). For instance, bacterial coinfections double mortality in COVID patients (Shah et al. 2023). Similarly, coinfecting helminths increased anaemia five‐to‐eight‐fold among children (Ezeamama et al. 2005). Coinfection at the within‐host scale can also influence population‐scale pathogen community structure (Ramesh et al. 2024) and disease dynamics (Ezenwa and Jolles 2011; Mideo et al. 2008). Populations with a higher frequency of coinfection experience larger epidemics than those with single‐species epidemics (Susi et al. 2015). Yet, despite virulent costs, coinfections are pervasive among humans, wildlife, and livestock/agricultural hosts (Ezenwa et al. 2010; Halliday et al. 2020). Therefore, we need to better understand why and how parasites coinfect their hosts.

**FIGURE 1:**
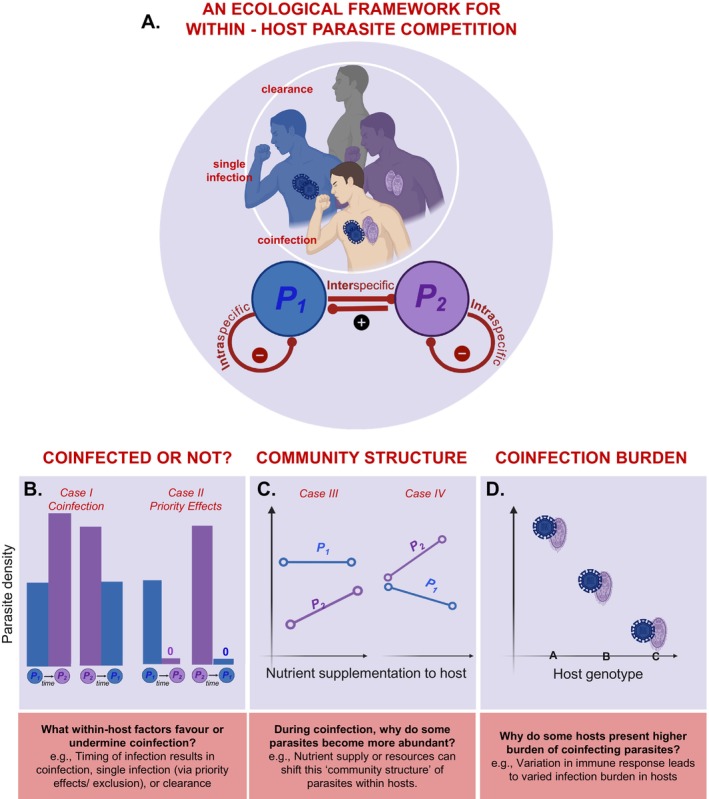
*Unpacking causes and consequences of coinfection*: (A) Exposure to multiple parasite species can lead to divergent outcomes captured by simple competition models, including coinfection, single infection, or clearance from hosts. Parasite competition hinges on how (a) species compete with themselves (intra‐specific competition: Negative direct effects, red curves), and how (b) species compete interspecifically (negative direct effects, red arrows). Generally, coinfection (or within‐host coexistence) occurs at an equilibrium promoting positive densities of both (‘feasible interior’) when intra‐ exceeds inter‐specific competition. In niche models, it manifests through niche factors like energy or immune cells. (B–D) Major questions emerging from two decades of coinfection experiments include: (B) *Coinfected or not?* What within‐host factors facilitate coinfection and what prevents it? Sequential exposure to parasite species can lead to coinfection irrespective of timing (Case I); in other scenarios, early infecting parasites prevail (impeding coinfection; priority effects, Case II). (C) *Coinfection community structure*: When they coinfect, why do some parasites become more abundant than others? When can nutrient supply or resources increase a parasite's relative abundance (Case III) and both relative and absolute abundance (Case IV)? (D) *Coinfection burden*: Finally, do differences in coinfection burden among hosts (or host genotypes) arise from variation in allocation to immune resistance?

Nonetheless, coinfection represents just one outcome of concurrent pathogenesis. That fact poses fundamental questions about coinfection. First, if coinfection is indeed detrimental to the host, then what within‐host factors facilitate it? Equally importantly, what prevents it? Within‐host parasite competition can lead to coinfection (i.e., within‐host parasite coexistence), single infection (through exclusion or priority effects), or clearance (no infection) from hosts (Figure 1A; Vogels et al. 2019). For instance, irrespective of timing, sequential exposure to some parasites always leads to coinfection, yet for others, only early arriving parasites prevail (Figure 1B; Clay et al. 2019; Devevey et al. 2015). Second, when they coinfect, why do some parasite species increase in abundance relative to others? Notably, nutrient supply or resources can shift ‘community structure’ of parasites within hosts (Figure 1C; Fellous and Koella 2009; Budischak et al. 2015). For example, zinc nutrient supplementation in children reduced *G. lamblia* infections but increased *A. lumbricoides* infections (Long et al. 2007). Finally, some hosts present a higher coinfection burden than others, hinting at variation in immunological resistance (Figure 1D; Fuess et al. 2021; Halliday et al. 2018). How can allocation to immune function govern coinfection burden? Presently, a key hurdle remains the lack of a mechanistic model that ties these disparate pieces together, with no synthetic glue to unify them into a coherent framework (but see Sofonea et al. 2017). Here, we seek to catalyse the creation of a framework linking the genesis of coinfection to its consequences (Lively et al. 2014; Restif and Graham 2015).

**FIGURE 2:**
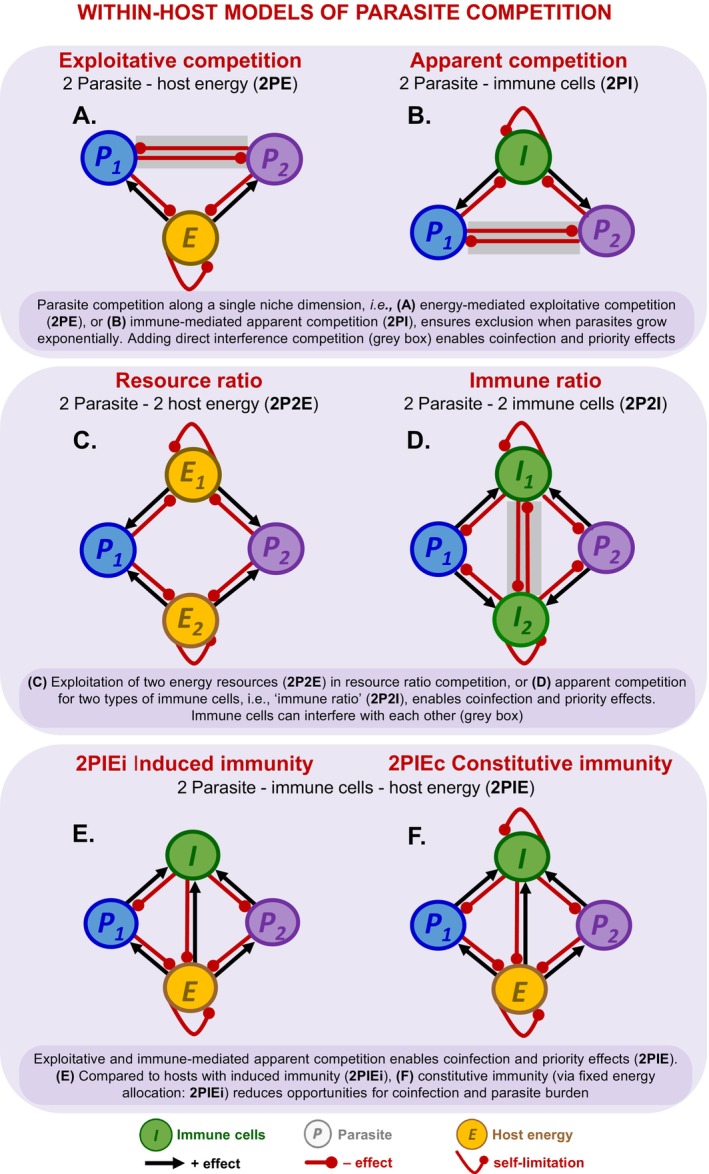
*Models of within‐host competition of parasites*, *with potential infection outcomes*. *Top row*, *single host energy or immune cells model*: Two parasites can compete indirectly for (A) host energy (exploitative: 2PE) or (B) via immune cells (apparent competition: 2PI). Parasites can also engage directly in interference competition (denoted by *P*_*1*_ *—P*_*2*_ interactions: Grey shading). *Middle row*, *two energy or immune models*: Two parasites can compete for (C) two sources of energy or other resources within hosts (2P2E; i.e., a resource ratio model) or (D) attacked by two different classes of immune cells (2P2I; ‘immune ratio’; mutual inhibition between *I*_*1*_ *—I*_*2*_ is possible [grey shading]). *Bottom row*, *energy and immune models*: Competing parasites can be attacked by shared immune cells and compete for host energy with (E) induced immunity (2PIEi), and (F) with constitutive immunity (2PIEc). Immune cells require host energy for proliferation resulting in additional *E‐I* links beyond those above (B, D). Red (black) arrows: Negative (positive) interspecific direct effects, evaluated at positive densities (a ‘feasible interior’ equilibrium); red curved arrow: Self‐limitation (negative intraspecific effect).

**FIGURE 3:**
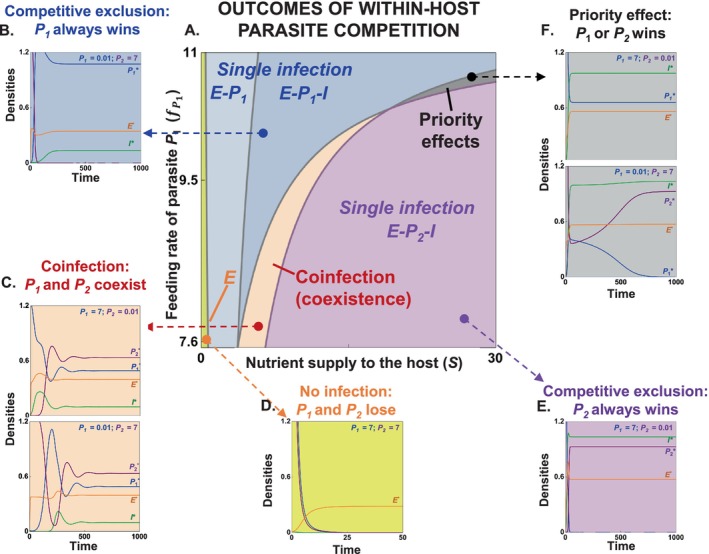
*Outcomes of within‐host competition in the 2PIEi model* (A) The range of competition outcomes are summarised in a 2D‐bifurcation diagram over gradients of nutrient supply (*S*) and feeding rate of parasite *P*_*1*_ ($f_{P_{1}}$). The (a)symmetry in inter‐ and intra‐specific competition involved governs single infection (via competitive exclusion: *P*_*1*_ [blue] or *P*_*2*_ [purple] only), coinfection (orange), priority effects (grey), or no infection (yellow). (B—F) Sample dynamics for one or two starting densities of *P*_*1*_ and *P*_*2*_. Feeding rate of *P*_*2*_ fixed here on ($f_{P_{2}}$ = 10.63; all values in Table S1).

**FIGURE 4:**
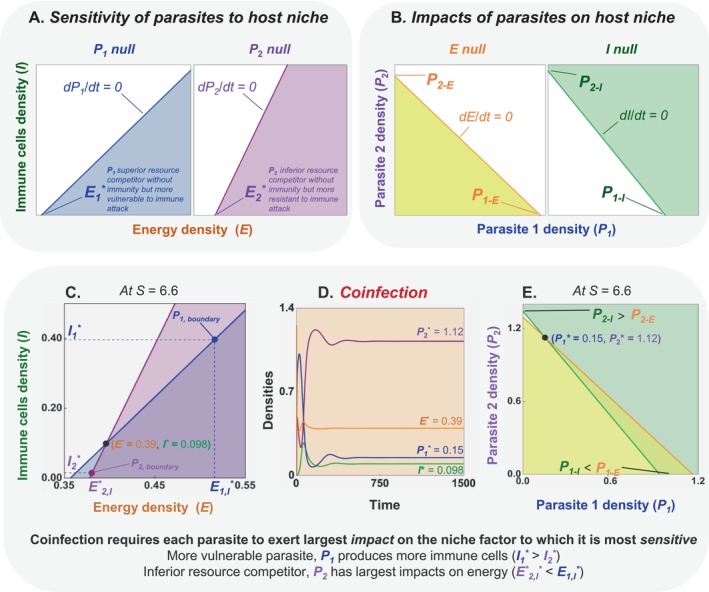
*Interpretation of coinfection (coexistence) in the 2PIEi model using nullclines*. (A) *Sensitivity to their niche*: Densities of energy (*E*) and immune cells (*I*) at which parasites 1 (*P*_*1*_) and 2 (*P*_*2*_) show zero growth (*dP*_*j*_/dt = 0). Combinations below these nullclines (higher energy, less immune attack) fall in the fundamental niche of each parasite. (B) *Impacts on their niche*: Densities of parasite 1 (*P*_*1*_) and 2 (*P*_*2*_) below the energy nullcline (*dE*/dt = 0, yellow) or above the immune nullcline (*dI*/dt = 0, green) lead to increases in both. At a given *S*, the outcomes of within‐host competition can also be visualised at the intersection of (C) parasite or (E) energy‐immune cell nullclines involving the (D) equilibrium denoting coinfection (●) (See text and Appendix A, Tables S1 and S2 for more details).

**FIGURE 5:**
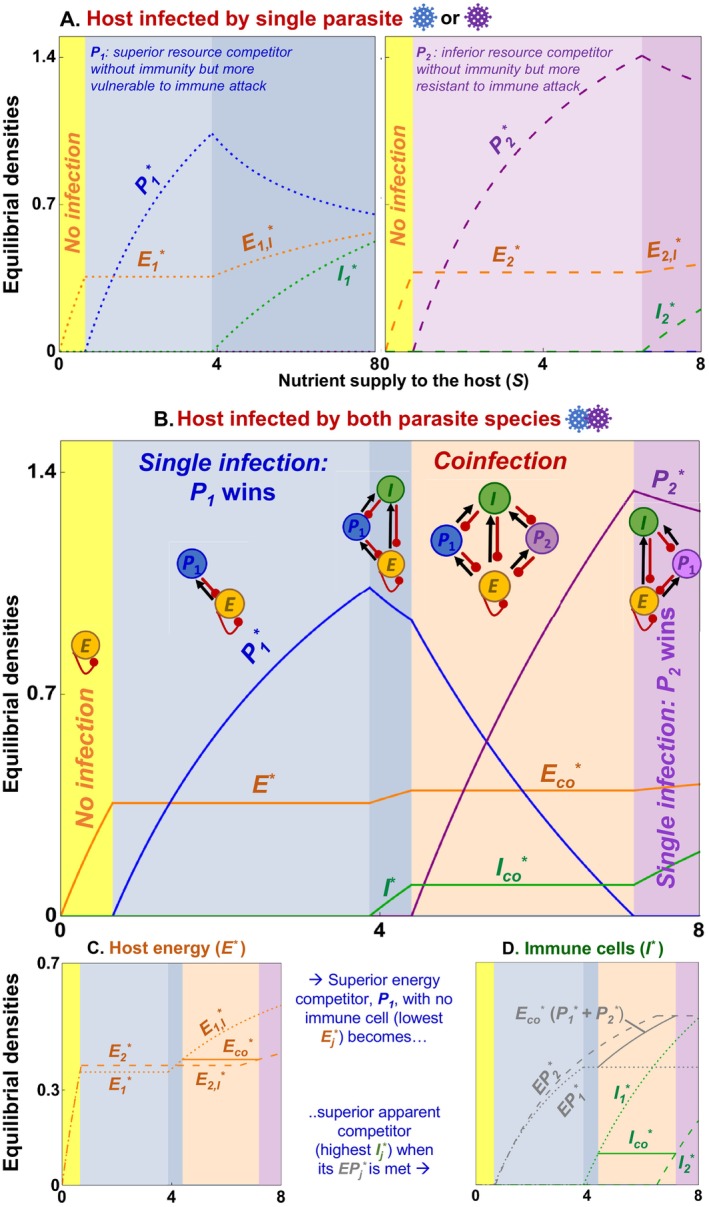
*Elements of coinfection (coexistence) in the 2PIEi model along gradients of nutrient supply*. (A, B) Equilibrial densities along a gradient of nutrient supply (*S*) of (A) one single parasite, *P*_*1*_ (blue *……*) or the other, *P*_2_ (purple—–) or (B) by both parasites (coinfection in orange; $f_{P_{1}}=7.2;f_{P_{2}}=10.63$). Energy (*E*) and immune (*I*) quantities of each parasite influences outcomes of joint resource (energy) and apparent competition. (C) *Energy competition*: Without immune attack, *P*_*1*_ is the superior energy competitor (*E*_1_* < *E*_2_*) allowing *P*_*1*_ to win (*sensu* the R* rule). Mortality from immune activation weaken *P*_*1*_'s competitive ability, allowing *P*_*2*_ to become the superior energy competitor (*E*_*1*,I_* > *E*_*2*,I_*). (D) *Immune‐ mediated apparent competition*: Immune cells proliferate when their minimum energy‐parasite requirement is met (*EP*_1_*: Grey *……*; *EP*_2_*:—–). The winner of apparent competition (higher *I*_j_*) supports more immune cells (here: Less immune resistant *P*_*1*_). (See text and Appendix A, Tables S1 and S2 for more details).

**FIGURE 6:**
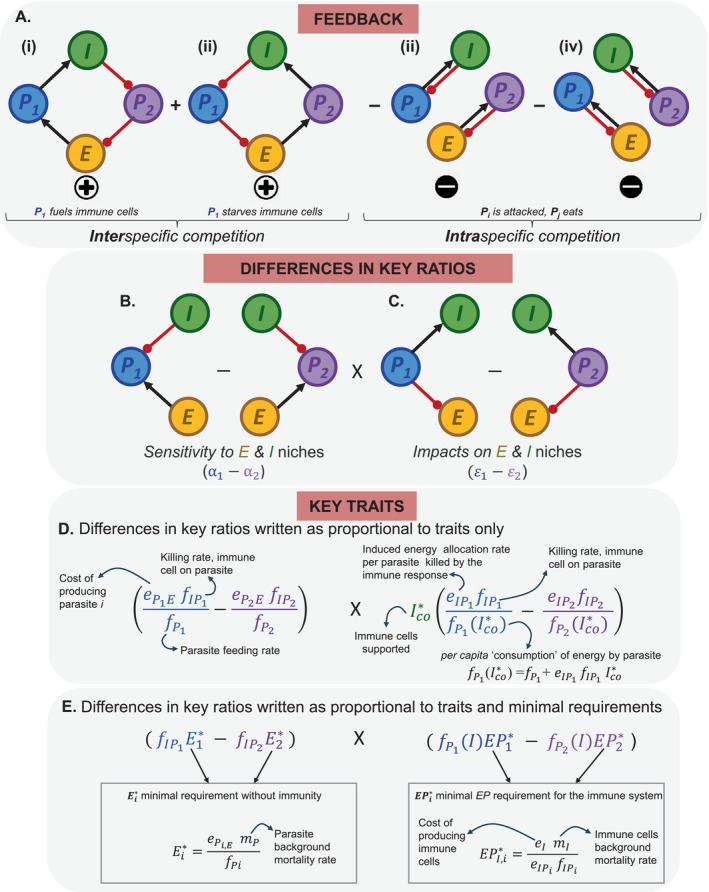
*Links between feedback loops*, *sensitivity and impact ratios and key traits*. (A) *Feedback*: Coinfection *v* priority effects is governed by loops through the two parasites and two niche factors; from *L‐R*, two destabilising, positive (+), interspecific competition loops and two stabilising, negative (−), intraspecific competition ones. *P*_*i*_ benefits from an increase in its density (positive feedback, interspecific) as it (i) directly “*fuels*” or (ii) indirectly “*starves*” immune cells but is restrained (negative feedback, intraspecific) by (iii) and (iv) “*P*_*i*_ *is attacked*, *P*_*j*_ *eats*” loops (the product of binary *I‐P*_*i*_ and *P*_*j*_ *‐E* consumer‐resource‐like loops). (B) *Differences in ratios of key traits*: With some rearrangement, these feedback loops correspond to ratios of key quantities made of key traits. These ratios encapsulate differences in how competing parasites are (B) *sensitive to* (*α*_1_—*α*_2_) and have (C) *impacts on* (*ε*_1_—*ε*_2_) immune cells and energy. Those differences in ratios can be written proportional to and measured either as a combination of (D) traits or as (E) traits and minimal requirements. For instance, (E) *α*_*j*_ is proportional to the product of killing rate of immune cell on parasite *j* ($f_{{\mathrm{IP}}_{j}}$) and the minimal energy requirement of parasite *j* (*E*_j_*), and *ε*_*j*_ is proportional to the per capita ‘consumption’ of energy by parasite *j*, $f_{P_{j}}\left(I\right)$, times minimal energy and parasite needed for immune activation (*EP*_j_*).

**FIGURE 7:**
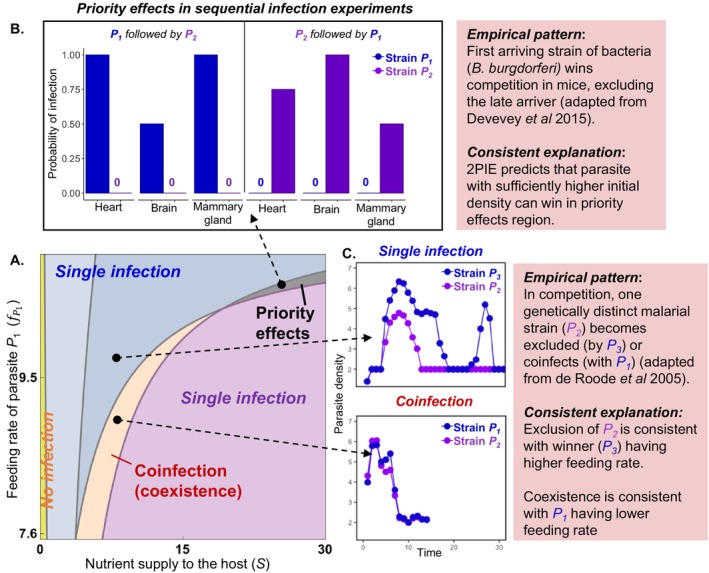
*Coinfected or not?* Assuming a competition‐resistance trade‐off between competitors, (A) the 2D bifurcation plot of resource supply to host resources (*S*) and feeding rate of parasite *P*_*1*_ on host energy ($f_{P_{1}}$) captures divergent infection outcomes in 2PIEi (as in Figure 3): Single infection either via priority effects (grey), or competitive exclusion (blue: *P*_*1*_ wins; purple: *P*_*2*_ wins), coinfection (orange), or no infection (yellow). (B) *Priority effects* in mice parasitized by strains of a bacterium ( *B. burgdorferi* ), where the first invader excludes the later. (C) *Competitive exclusion*: A malarial strain (*P*_*2*_; purple) is excluded by one (*P*_*3*_) or *coinfects* (coexists) with another strain (*P*_*1*_; blue).

**FIGURE 8:**
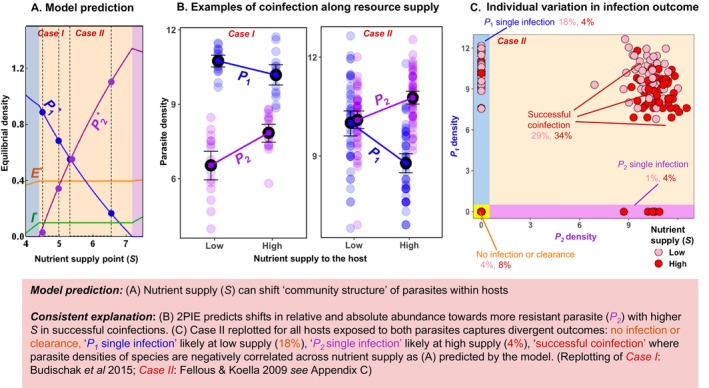
*Coinfection community structure*: Coinfecting parasites can shift relative and absolute abundance within hosts with increasing resource supply (*S*). (A) *Model*: In 2PIEi, increasing nutrient supply favours more resistant *P*_*2*_ over the superior *P*_*1*_. (B) *Experiments*: Empirically, shifts in community structure arise in mice coinfected by two species of gastrointestinal worms (case I) and a mosquito larva infected by a microsporidian and protozoan (case II). (C) *Extension*: Re‐examination of all results from Case II suggests that variation in infection outcomes could arise when individuals assimilate different amounts of food. Small deviations among individuals could drive large variation in infection outcomes.

**FIGURE 9:**
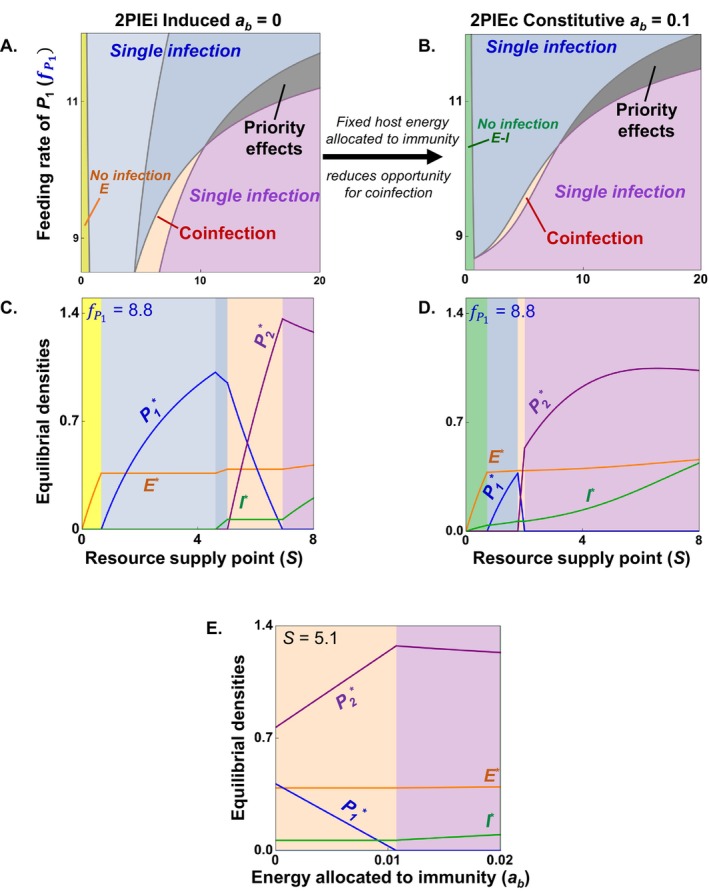
*Coinfection burden*: The burden of parasites that infect a host can depend on baseline energy allocation to immunity (*a*_*b*_). (A) vs. (B): Higher *a*_*b*_ squeezes parameter space for coinfection (2PIEi, induced *v* 2PIEc, constitutive immunity, respectively). (C, D) It also reduces the density of competing parasites. Reduced burden allows the host to maintain slightly more energy for other metabolic work, potentially improving host health. (E) Increasing *a*_*b*_ favours the superior energy competitor (more immune resistant *P*_*2*_) over the superior apparent competitor (*P*_*1*_), eventually excluding it. By implication, differences in *a*_*b*_ can lead to varying (co)infection burdens among hosts.

A mechanistic, within‐host framework of parasite competition provides a start. To illustrate, we develop and evaluate within‐host models of coinfection, synthesising insights through an ecological lens (following Ramesh and Hall 2023). Specifically, we advocate the conceptualisation of coinfection as coexistence among parasite species (Figure 1A). Generally, coinfection occurs when each parasite species competes more strongly with itself (intraspecifically) than with each other (interspecifically). Those competitive differences could be measured phenomenologically (Gruner et al. 2009). Even better, they can be quantified from trait‐based niche models for parasites competing within hosts (Graham 2008; Cressler et al. 2014). In such models, parasites interact within host ‘ecosystems’ for shared resources while facing attack by energetically costly immune cells. Simple ecological rules, then, govern within‐host parasite competition. To illustrate, we first borrow from older niche models to outline new models of competition for shared energy (resources) and/or immune cells among parasites (Figure 2). Second, using a detailed study of a model of two parasites, immune cells, and energy (2PIE), we link outcomes of infection to competitive abilities, nullclines (niches), feedback, and key traits (Figures 3, 4, 5, 6). Third, we demonstrate how a priori predictions from these models can be linked to the interpretation of experimental outcomes (Figures 7, 8, 9). Finally, we offer suggestions for future theory and experiments (Table 1). Taken together, we use a within‐host competition model to lay the foundational principles for understanding the cause and consequences of parasite coinfection via a niche framework.

## 2 A Phenomenological Approach to Coinfection

The two‐species Lotka–Volterra model of competition makes testable predictions for within‐host competition of parasites (Lotka 1925; Volterra 1926). Like species coexistence, successful coinfection requires that each species exerts a stronger negative effect on its own per capita growth rate (fitness) than on its competitor's, i.e., intraspecific exceeds interspecific competition creating stabilising negative feedback (Figure 1A). Coinfection is hindered in one of two ways. First, priority effects arises if each species more strongly affects its competitor's fitness than its own; i.e., inter‐ exceeds intra‐specific competition. Priority effects typically give rise to two alternative states, depending on the initial density of each parasite. The resulting positive feedback leads to single infection by one or the other. Second, competitive exclusion arises with asymmetric competition, where only one species can invade its competitor's single‐species state (‘boundary equilibrium’) but not vice‐versa. Hence, mutually or asymmetrically strong interspecific competition can hinder coinfection. Empirically, one can derive estimates of competition coefficients from experiments that manipulate parasite densities and measure growth rates, including via a response surface design. This design involves growing parasite species alone or at minimal densities that lead to infection, as well as with increasing densities of intra‐ and interspecific competitors (Table 1A). It allows estimation of coefficients of competition to predict species infection success when rare (Freckleton and Watkinson 2000; Hart et al. 2018). However, this phenomenological approach makes inferences about a specific environment, limiting predictions of infection outcomes across gradients of traits, nutrient supply, and/or allocation to immunity. Such contexts require a mechanistic framework involving host immunity and/or host resources.

## 3 Within‐Host Feedbacks Driving Divergent Infection Outcomes

To move toward such a framework, we outline six models of within‐host competition between parasites involving immunity, resources, and/or direct interference. To visualise these interactions, we label the direct effect of species *j* on the growth rate of species *i*, yielding (hereafter) interspecific *positive* (black arrow) and *negative effects* (red) and intraspecific *self‐limitation* (red curve; Figure 2). All models potentially contain mechanisms for coinfection, single infection due to exclusion or priority effects (aka, alternative states or founder control), and no infection. Although details vary, such divergent outcomes follow general principles of competition (above). In essence, coinfection occurs when intraspecific competition (direct or looped indirectly via ‘niche’ factors like resources or immune cells) outweighs interspecific competition at an equilibrium supporting positive densities of all (a ‘feasible interior’). Here, stronger intraspecific competition leads to net negative feedback, facilitating stable coinfection.

### 3.1 Two Parasite—Shared Energy (2PE; Exploitative) or—Shared Immune Cells (2PI; Apparent Competition)

Parasite species that share a resource (energy) or suffer attack from a shared immune system engage in exploitative or apparent competition, respectively. In exploitative competition (Figure 2A), a parasite that can survive at a lower equilibrium energy (resource) level (E*) wins. For instance, among two clones of a rodent malaria (*P. chabaudi*), the superior competitor for red blood cells (RBC) excludes the inferior clone (De Roode et al. 2005). Competition for a single, shared resource can lead to a competitive hierarchy among multiple parasite species, e.g., one Plasmodium species (*P. falciparum*) outcompetes hookworms, which themselves outcompete another Plasmodium ( *P. vivax* ) for red blood cells (Budischak et al. 2018). Analogous rules can apply to parasites sharing an immune system (Figure 2B). In models with two exponentially growing parasites, the species that withstands the highest density of immune cells (i.e., highest I*) wins via apparent competition (Fenton and Perkins 2010). In a possibly analogous experiment, immune‐mediated interactions led to competitive suppression of an avirulent malarial clone, allowing the virulent clone to dominate (Råberg et al. 2006). Therefore, exploitative competition for a single resource or apparent competition involving shared immune cells can produce competitive exclusion.

Two aspects of within‐host parasite niches can introduce coinfection or priority effects. First, if both parasites experience direct self‐limitation (rather than indirectly through their niche), they can coinfect even when they share one immune niche. For instance, parasites experience self‐limitation when they solely recruit via ‘immigration’ onto hosts via propagule influx (esp. macroparasites) and when immune clearance is non‐linear, particularly when it accelerates at higher parasite density (Fenton and Perkins 2010). Second, interference competition between parasites can facilitate priority effects or coinfection (Amarasekare 2002; Figure 2A,B shows mutually inhibitory effects between *P*_*1*_ and *P*_*2*_ [grey shading]). If the inferior energy competitor is superior at interference, it can win via priority effects (rather than face exclusion) with high enough initial densities. However, if interference also benefits the interacting species (e.g., if killing competitor larvae and consuming it increases *per capita* growth rate of the competitor), then coinfection ensues (Amarasekare 2002). These principles for joint interference and exploitative competition likely also apply to immune‐mediated apparent competition, a possibility awaiting future exploration.

### 3.2 Two parasite—two energy (2P2E; Resource ratio) or Two parasite—two immune cells (2P2I; Immune ratio)

When parasites (*P*_*j*_) simultaneously compete for two resource or energy sources (*E*_*i*_) via exploitative competition, infection outcomes can include coinfection and priority effects. Consider two parasite species competing for two substitutable resources (like in the resource ratio model [Tilman 1982]: Figure 2C). Here, coinfection minimally requires each species to trade off their requirements for each energy resource that is, each parasite uses energy sources *E*_*1*_ more effectively than *E*_*2*_ and vice versa. Then sufficiently intermediate supply ratios must permit each single‐species (‘boundary’) equilibria to fall within each competitor's niches enabling mutual invasibility. With both conditions met, coinfection arises if each parasite has a larger impact on the resource to which its fitness is most sensitive (see Appendix Section 2). Lacroix et al. (2014) tested the resource ratio model and found a clear competitive hierarchy in plants co‐inoculated with virus species. For instance, at low nitrogen: phosphorus (N:P) supply, the superior *N* virus competitor enabled higher self‐replication rates, reducing the density of its competitor within their plant host (Lacroix et al. 2014; Smith 2014). These outcomes suggest that the N:P ratio shapes how each parasite species differentially responds to and impacts their resources.

Analogous rules likely apply to hosts with two types of immune response (2P2I, the immune ratio model; Figure 2D). If parasites act as substitutable ‘resources’ for two generalist immune responses, coinfection arises if each parasite also has a larger impact on the immune response to which its' fitness is most sensitive (Appendix B). Modification of 2P2I captures several within‐host interactions. First, the host can mount an independent, specialist immune response to each parasite (Fenton and Perkins 2010). Therefore, the separate dynamics of each immune‐parasite pair determine coinfection. Similar results emerge for parasites separated spatially (e.g., in different host tissues: Cervi et al. 2004; Karvonen et al. 2006). Second, two parasites can each be attacked by specialist immune responses that inhibit each other. For instance, in mice, parasites interact via T‐helper cells, where Th1 attacks intra‐cellular malaria, Th2 attacks intestinal nematodes, but Th1 and Th2 inhibit each other (Griffiths et al. 2015). Such interference creates negative feedback that can enable coinfection (e.g., links between *I*_*1*_ and *I*_*2*_ [grey]; Fenton and Perkins 2010).

### 3.3 Two Parasite—Immune Cell—Energy (2PIE)

Parasites can also engage in simultaneous exploitative and immune‐mediated apparent competition (Cressler et al. 2014; Ramesh and Hall 2023). Consider common variations of two competing parasites (*P*_*j*_) that steal energy (*E*) from hosts and share immune cells (*I*) that kill them (2PIE; Figure 2E,F; Table S1). In one, only parasites induce the production of immune cells (2PIEi, induced immunity). In the other, energy is continuously allocated to maintain baseline immune function, even without parasites (2PIEc, constitutive immunity). This structure resembles a food web in which two prey share a resource and a predator (Holt et al. 1994; Leibold 1996). This keystone predation (diamond) model anticipates how trade‐offs and niche dimensions govern exploitative and apparent competition between parasites (Ramesh and Hall 2023). When combined, these forms of competition enable coinfection, priority effects, or competitive exclusion (see Figure 3; Ramesh and Hall 2023). Constitutive immunity (Figure 2F) reduces opportunities (parameter space) for coinfection, lowering parasite burden while maintaining higher host energy (see below; Figure 9).

The remainder of this synthesis will focus on joint immune and energy (resource) competition in the 2PIE model. Most organisms have (costly) immune defences to fight parasites that steal host energy (*reviewed in* Lochmiller and Deerenberg 2000; Zuk and Stoehr 2002). Furthermore, principles that govern intra‐ *v* inter‐specific competition in 2PIE models should apply to others. Building on previous analogies to competition in food webs (Ramesh and Hall 2023), we synthesise how resource and apparent competition underpin a within‐host framework. This framework connects nullclines (niches), competitive abilities, feedbacks, traits, and resulting infection outcomes. With the 2PIE model, then, we ask: (a) What within‐host feedback drives outcomes of infection? (b) Can this framework offer new interpretations of previous coinfection experiments? (c) How can this framework guide future theory and experiments?

## 4 A Mechanistic Framework Linking Feedbacks, Trait Ratios, and Infection Outcomes

In this section, we develop 2PIE. It follows Lotka‐Volterra principles (above), where intra‐ vs. inter‐specific competition governs coexistence vs. priority effects (Figure 1A). Although specifics differ between 2PIEi and the others (Figure 2A–F), this case study generates mechanistic a priori predictions and insights into coinfection dynamics from within‐host niche models (algebra in Appendix A).

### 4.1 The Model

*Growth rate of immune cells I* (Equation 1A): In 2PIEi, immune cells (*I*) increase after attack on parasite *j* (*P*_*j*_) at rate $\;f_{IP_{j}}$, inducing consumption of $e_{IP_{j}}$ of energy, *E*, with conversion efficiency *e*_*I*_ of energy into an immune cell and loss $m_{I}$. In 2PIEc, *I* also increases with baseline allocation of *E* at rate *a*_*b*_.

*Growth rate of parasite P*_*j*_ (Equation 1B): The two parasites consume host energy with feeding rate $f_{P_{j}}$ and energy per parasite conversion, $e_{P_{j}E}$ ^−1^. The parasites are lost due to attack by immune cells ($\;f_{IP_{j}}$) and die at (shared) background rate $m_{P}$.

*Growth rate of energy*, *E* (Equation 1C): The host consumes resource *S* via a Monod function with maximal assimilation rate $f_{E}$ and half‐saturation constant *h*. (This *f*(*S*) function pays homage to non‐linear feeding behaviour of hosts: Cressler et al. 2014). That resource, converted to energy (*E*), is lost at fixed rate *r* for metabolic use by hosts. Its net production, then, is *f*(*S*) – *r E*. Additionally, host energy is consumed via induction from immune attack on parasites (proportional to *P*_*j*_ *E I*), and from consumption (theft) by parasites. Hence, parasite *j* ‘consumes’ energy both indirect and directly, grouped as *f*_*j*_(*I*). In 2PIEc, energy is allocated to baseline immune function at rate *a*_*b*_. The 2PIEi,c models are thus (see also Appendix A, Table S1):

(1A) $$\frac{\mathrm{dI}\;}{\mathrm{dt}}=\frac{a_{b}E+\Sigma \;e_{IP_{j}}f_{IP_{j}}\;P_{j}E\;I}{e_{I}}-m_{I}I$$

(1B) $$\frac{dP_{j}}{\mathrm{dt}}=\left(\frac{f_{P_{j}}\;E}{e_{P_{j}E}}-f_{IP_{j}}I-m_{P}\right)\;P_{j}$$

(1C)

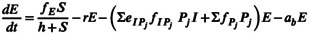




### 4.2 Outcomes of Competition

We map predictions of 2PIEi along gradients of resource supply to hosts, *S*, and feeding rate of parasites on energy, $f_{P_{1}}$ (Figure 3A, Table S2). The lines on this map (a 2D bifurcation diagram) represent qualitative compositional shifts determined by minimal requirements as detailed in the next section. Depending on $f_{P_{1}}$ and *S*, competing parasites can produce no infection (yellow), single infection via competitive exclusion (*P*_*1*_ wins: blue shades; *P*_*2*_ wins: purple shades), successful coinfection (orange), or priority effects aka founder control (grey). The offsets show dynamics within hosts concurrently exposed, started at one or two initial densities (Figure 3B–F). In the no infection region, insufficient energy led to failed infection, despite high initial densities of parasites (nutritional clearance; Figure 3D). In single infection regions, the winner always excludes the other, even starting at low densities (Figure 3B,E). In the coinfection region, all trajectories head to a stable equilibrium (Figure 3C). With priority effects, the one starting more densely wins and excludes the other (i.e., top: *P*_*1*_ wins; bottom: *P*_*2*_; Figure 3F).

### 4.3 Mechanism of Competition: A Nullcline and Within‐Host ‘Assembly Rules’ Approach

The outcomes in 2PIEi hinge on intra‐ vs. inter‐specific competition. In general, intraspecific competition, mediated indirectly via host energy and immune cells, produces negative feedback loops while interspecific competition yields positive ones (Figure 1A). When the strength of intra‐ exceeds inter‐specific competition, net negative feedback on the feasible interior equilibrium can enable coinfection. Conversely, greater inter‐ than intra‐specific competition generates net positive feedback that can produce priority effects. This section actualises these concepts for 2PIEi using both nullclines (a visualisation of niches) and construction of within‐host ‘assembly’ rules. While nullclines establish three conditions for coinfection, assembly rules reveal why and how these relationships arise along a nutrient gradient.

#### 4.3.1 A Nullcline (Niche) Interpretation

Nullclines characterise the niche environment for coinfection, showcasing both sensitivity to and impacts of each parasite species on energy and immune cells. After showing each nullcline separately (Figure 4A,B), we combine them to illustrate coinfection (Figure 4B–E).

##### 4.3.1.1 Single Infection

*Sensitivity* (Figure 4A): At a given nutrient supply *S*, nullclines denote conditions in which a parasite (*P*_*j*_) or niche component (energy, immune cells) neither grows nor declines. Combinations of energy (*E*) and immune (*I*) cells that fall to the right of a parasite's nullcline (higher energy, lower immune cells) sit within its fundamental niche (*P*_*1*_, blue shading; *P*_*2*_, purple shading). The single species boundaries provide insights into minimum resource requirements of parasites for energy (*E*_j_*, akin to R* *sensu* Tilman 1982) and highlight maximal immune cell densities supported by each parasite (*I*_j_*, akin to P* *sensu* Holt 1977). While both parasite species follow similar nullcline patterns, they differ in a key trade‐off: *P*_*1*_ is the superior resource competitor without immune cells (x‐axis: *E*_1_* < *E*_2_*) but is more vulnerable to immune attack; i.e., for a given value of *E*, *P*_*2*_ can persist at higher values of *I*. That competition‐resistance trade‐off provides a first prerequisite for coinfection.

*Impacts* (Figure 4B): Then, the impact of each parasite on its within‐host niche determines *I* and *E* nullclines. When coinfected, the nullcline for *E* is the combination of *P*_*1*_ and *P*_*2*_ that ‘consumes’ all production of energy at the interior (*E*_co_*). Below this line, energy grows (d*E*/d*t* > 0, yellow). When singly infected, the intercepts (*P*_*j‐E*_) denote the density of each parasite that consumes all net energy production. Here, *P*_*2*_ consumes all energy at a slightly lower density than does *P*_*1*_ (*P*_*2‐E*_ < *P*_*1‐E*_).

The nullcline for *I* centres on immune activation. This activation requires a sufficient product of host energy and parasite densities, a minimum ‘resource’ requirement of immune cells (*EP*_j_*). When coinfected, the nullcline for *I* combines *P*_*1*_ and *P*_*2*_ to meet the minimal EP* for immune activation given energy set at *E*_co_*. All densities above the line increase the production of immune cells (d*I*/d*t* > 0; green). The intercepts (*P*_*j‐I*_) note the density of each parasite alone that supports immune activation (at *EP*_j_* given *E*_co_*). Here, *P*_*1*_ produces more immune cells at a slightly lower density than does *P*_*2*_ (*P*_*1‐I*_ < *P*_*2‐I*_).

##### 4.3.1.2 Coinfection

*Coinfection* (● in Figure 4C–E): Coinfection requires that parasite nullclines cross, niche nullclines cross, and nutrient supply is intermediate. The nullclines of the parasites cross in *E–I* space because of the competition‐resistance trade‐off. The shallower slope of less immune resistant *P*_*1*_‘s nullcline reflects higher sensitivity to *I*; in contrast, the steeper nullcline for *P*_*2*_ indicates higher sensitivity to *E* (Figure 4C). At the crossing of nullclines (●), coinfecting parasites set *E* and *I* (Figure 4D). When they coinfect, the single‐species (‘boundary’) equilibrium of one parasite falls within the fundamental niche of the other (i.e., the *P*_*1*_ equilibrium (blue dot) falls within *P*_*2*_‘s niche (purple) and vice‐versa). Hence, each single‐parasite host environment can be invaded (mutual invasibility). When they coexist, *P*_*1*_ is the superior apparent competitor (*I*_1_* *> I*_2_*) while *P*_*2*_ is the superior energy competitor (i.e., lower E*: *E*_*1*,*I*_ * *> E*_*2*,*I*_ *; Figure 4C).

Meanwhile, the *I* nullcline is steeper than the *E* nullcline in *P*_*1*_ *–P*_*2*_ space (Figure 4E). Hence, *P*_1_ has a larger impact on immune cells, while *P*_*2*_ has a larger impact on energy. Therefore, when they coinfect, each parasite exerts a larger impact on the niche factor to which it is most sensitive (Figure 4C). The *E* nullcline intercepts also depend on nutrient supply. If nutrient supply is intermediate (as shown), *I* and *E* nullclines cross interiorly. Here, the host environment would support surplus *P*_*1*_ (*P*_*1‐I*_ *< P*_*1‐E*_) but deficient *P*_*2*_ (so *P*_*2‐I*_ > *P*_*2‐E*_) than needed to meet the immune system‘s EP* requirement (Figure 4E). The coinfection equilibrium, then, combines densities of the superior apparent (*P*_*1*_) and energy competitor (*P*_*2*_) that consumes all net energy production while meeting the requirements of immune cells—eliminating those surpluses and deficits.

#### 4.3.2 Within‐Host ‘Community Assembly’ Rules

Several minimal requirements enabling coexistence (as shown in the nullclines) change along the host's nutrient supply gradient (*S*). We visualise these shifts—and the assembly rules that follow—using plots from single‐ (Figure 5A, ······ or – – –) and two‐parasite cases (Figure 5B, —).

*Single infection* (Figure 5A): Single infection follows resource‐ and immune‐based assembly rules along a nutrient supply gradient, *S*. Very low nutrient supply (*S*) meets neither parasite's minimum energy requirements (E* < *E*_j_*). Hence, very low *S* prevents infection (i.e., clearance via starvation; yellow). However, with high enough *S*, E* increases, eventually meeting that minimum (*E*_j_*). Then, *P*_*j*_ can invade and increase with *S* (pinning *E* at *E*_j_*; *E‐P*_*j*_ regions in lighter). Eventually, *P*_*j*_'s density crosses a minimal threshold (*EP*_j_*) that induces immune activation (darker colours). With higher *S* still, immune attack reduces the density of *P*_*j*_, freeing more energy (at *E*_*j*,I_*). As shown, *P*_*1*_ is the superior competitor without immune cells (*E*_1_* < *E*_2_*) but is more vulnerable to immune attack (echoing their nullcline orientation [Figure 4A]; Figure 5C).

*Route to coinfection* (Figure 5B): Given that competition‐resistance trade‐off, a multi‐parasite system undergoes four transitions along the nutrient gradient, *S*. First, more vulnerable *P*_*1*_ invades, excluding more immune‐resistant *P*_*2*_ via resource competition (since *E*_1_* < *E*_2_*). At some *S* following immune activation (*EP*_1_*), *P*_*1*_ becomes the superior apparent (*I*_1_* > *I*_2_*) but inferior energy (*E*_*2*,I_* < *E*_*1*,I_*) competitor. Then, with further *S*, the *E‐I* niche set by *P*_*1*_ enables *P*_*2*_ to invade. In a window of coinfection, the within‐host environment remains constant while parasite densities shift (Figure 5B). Here, energy and immune cells stay at *E*_co_* and *I*_co_*, respectively. However, with higher *S*, *P*_*2*_ increases while *P*_*1*_ declines. With this shift, the energy and parasite density needed to maintain immune activation, *E*_co_*(*P*_1_* + *P*_2_*), increases until *P*_*2*_ could support it alone (at *EP*_2_*; Figure 5D). In this region, the more vulnerable *P*_*1*_ remains the superior apparent competitor (Figure 5D, *I*_1_* *> I*_2_*) but inferior energy competitor (Figure 5C, *E*_2_* < *E*_*1*,I_* shifts to *E*_*2*,I_* < *E*_*1*,*I*_ *) and vice‐versa. Hence, the more vulnerable parasite produces more immune cells, the niche factor to which it is most sensitive. That combination brakes its growth rate, promoting coinfection. With higher *S*, *P*_*2*_ excludes *P*_*1*_ (via energy competition: *E*_*2*,I_* < *E*_*1*,I_*), but increasing immune cells lowers *P*_*2*_'s density (Figure 5B–D).

*Priority effects*: As detailed (Table S2), priority effects still require a competition‐resistance trade‐off, hence differential sensitivities to niche factors. However, now each parasite exerts a greater impact on the niche factor to which its competitor is most sensitive. Biologically, the more resistant species (*P*_2_) produces more immune cells to which their competitors are most sensitive. Simultaneously, the better energy competitor without immune cells (*P*_1_) remains superior with them, exerting a greater impact on energy to which *P*_2_ is most sensitive. These changes enable priority effects via positive feedback (see below).

#### 4.3.3 Factors Enabling Coinfection: Feedback Loops and Trade‐Offs of Traits

Outcomes of coinfection v priority effects also depend on feedback and traits that govern them. Most fundamentally, the within‐host environment must support co‐infection at an equilibrium having net negative feedback because intraspecific exceeds interspecific competition. This balance of competition depends on loops connecting parasites to their niche (Figure 6A, Appendix A). Such loops link increased density of a species to growth rates of others, eventually returning to that first species (Puccia and Levins 1991). For instance, interspecific competition can be traced starting with an increase in *P*_*1*_. (i) Higher *P*_*1*_ ‘*fuels immune cells’* that suppress its competitor, freeing up energy resources that *P*_*2*_ would consume. (ii) *P*_*1*_ also ‘*starves immune cells’* by consuming energy that depresses *P*_2_, hence lowering immune activation. Through both interaction chains, *P*_*1*_ indirectly benefits from an increase in its own density via gains in energy, hence birth rate, or via immune suppression, hence lower death rate. Those two positives (destabilising) loops then push against two negative loops, (iii and iv) “*P*_*i*_ *is attacked*, *P*_*j*_ *eats*”. These latter loops add stabilising, consumer‐resource‐like, intraspecific competitive interactions. For instance, (iii) a small increase in *P*_*1*_ increases immune attack, thereby reducing *P*_*1*_ (*P*_1_‐*I* loop), while *P*_*2*_ is slowed by resource consumption (*P*_*2*_‐*E* loop). Then, (iv) those roles reverse, i.e., resources brake *P*_*1*_ while the immune system slows parasite *P*_*2*_. Those two loops (iii + iv) jointly determine the amount of intraspecific competition (negative feedback). Hence, if the strength of intraspecific competition loops (iii + iv: negative) exceeds that of interspecific competition loops (i + ii: positive), net negative feedback leads to coinfection. If, instead, interspecific exceeds intraspecific loop strength, net positive feedback generates priority effects.

*Trait trade‐offs*: With some rearrangement, these intra‐ and inter‐specific loops governing feedback can connect to the niche environment and assembly rules above (Figure 6B,C). The rearrangement boils down to two quantities: the *sensitivity* and *impact* ratios, tightly linked to nullclines in Figure 4 (see Appendix A). Both quantities involve the difference in ratios of *sensitivity* of each parasite (Figure 6B), or its *impacts* on the immune cells, *I*, vs. energy, *E* (Figure 6C; following Pásztor et al. 2016). These ratios are:

- Sensitivity (*α*_*j*_), the rate of parasite killing due to immunity *v* turning energy into new parasite.
- Impact (*ε*_*j*_), the rate of immune stimulation by parasites *v* energy consumption by parasite *j*

Coinfection requires that each parasite has a larger impact on the niche factor to which it is most sensitive. If the more vulnerable *P*_*1*_ is more sensitive to and has a higher impact on *I*, then *α*_*1*_ > *α*_*2*_ and *ε*_*1*_ > *ε*_*2*_. Such symmetry in ratios generates net negative feedback. For priority effects, the competition‐resistance trade‐off still yields *α*_*1*_ > *α*_*2*_. However, because *P*_*1*_ has a higher impact on *E* (to which *P*_*2*_ is more sensitive) and *P*_2_ most impacts *I*, the impact ratios flip, *ε*_*1*_ < *ε*_*2*_. This asymmetry in ratios produces net positive feedback. Both ratios are proportional to the ratios of traits or traits and minimal requirements (*see* Appendix A).

*Quantifying traits* (Figure 6D): The (a)symmetry of the *sensitivity v impact* ratios governing coinfection and priority effects reflect differing combinations of key traits. For instance, the *sensitivity* ratios are governed by killing rate of immune cells on parasites ($f_{{\mathrm{IP}}_{j}}$), feeding rate of parasites ($f_{P_{j}}$), and the cost of producing a new parasite ($e_{P_{j}E}$). *Impact* ratios depend on how attacked parasites induce energy allocation to immune cells ($e_{{\mathrm{IP}}_{j}}f_{{\mathrm{IP}}_{j}}$) and direct and indirect ‘consumption’ of energy by parasite *j* ($f_{P_{j}}\left(I\right)$).

*Quantifying key traits and minimal requirements* (Figure 6E): Experimental tests of *sensitivity‐impact* ratios could also centre on measurement of key quantities such as the minimal requirement of each parasite (*E*_j_*) for energy and of the immune system for activation (*EP*_j_*; Figure 7E, Table 1C). For instance, the difference in sensitivity ratios boils down to killing rate of immune cells on parasites, $f_{P_{j}}$, and each parasite's minimal energy requirement without immune cells, *E*_j_*. Similarly, the difference in impact ratios depend on per capita ‘consumption’ of energy by parasites, $f_{P_{j}}\left(I\right)$, and the immune cells' minimal energy‐parasite requirement *EP*_j_* (Appendix A). These traits and quantities could, in principle, be estimated experimentally.

## 5 A Focus on the Traits and Quantities to Measure in the Future

Presently, most within‐host experiments observe patterns of coinfection, then infer mechanisms. The next phase of experimentation should link to feedback, minimal requirements, and traits of mechanistic niche models (like 2PIEi). Future research on coinfection dynamics could aim to quantify the traits and minima's underlying these sensitivity and impact frameworks. Despite calling for focus on such quantities, we acknowledge inherent challenges in measuring them, and we offer some thoughts looking forward.

First, an interdisciplinary approach may be needed to deduce the components of energy and immunity that govern within‐host dynamics. It is more straightforward to isolate these interactions in some systems. For instance, resource ratio models may apply to plant pathogenesis shaped by competition for multiple resources (Lacroix et al. 2014). In contrast, parasitism in honeybees may mostly depend on a single immune component or energy source (Moret and Schmid‐Hempel 2000). But what happens with competition under multiple immune arms or resources, as in vertebrate systems? In such cases, network or meta‐analysis may help pull out generalities (Graham 2008). For example, a network analysis of co‐infecting parasites showed that grouping by shared resources resulted in simplified model structures (like 2PIE: Griffiths et al. 2014; Rynkiewicz et al. 2015).

Second, with sufficient knowledge of baseline interactions, we can combine and add to simpler models. For example, the addition of interference competition to the 2PIE framework could better fit coinfection in mice, where malaria and nematodes compete for red blood cells and interact through multiple immune responses (Griffiths et al. 2015). In this case, Th1 clears malaria, Th2 clears nematodes, but each inhibits the other (Cressler et al. 2024), potentially creating stronger negative feedback that facilitates coinfection. Extending niche models to include these interactions could improve predictions of coinfection outcomes.

Finally, to move forward, experimentalists can leverage interdisciplinary methodologies. For instance, measurement of state variables like parasite (*P*_j_*), energy (*E*_j_*), or immune densities (*I*_j_*) over time can allow the estimation of key traits. Alternatively, measurements could follow radioisotope labelling of resources to track parasite feeding rate (LC–MS; Gomez‐Amaro et al. 2015), live imaging to track immune killing rate (Galli et al. 2021), ICP‐MS measurements of the energetic content of the infected host (Cassat et al. 2018), etc. Other studies have proposed mechanisms, with direct manipulation of in vivo resources and immunity demonstrating how an RBC‐generalist parasite can facilitate the replication of an RBC specialist (Ramiro et al. 2016). As a pay‐off, the combination of trait measurements with models generates a priori predictions of infection outcomes and the feedback mechanisms that govern them (notably demonstrated in Budischak et al. 2015; Griffiths et al. 2015). Ultimately, such approaches may catalyse a new wave of theory‐grounded, niche‐mechanistic combinations of modelling and experimentation.

## 6 Explanations for Divergent Infection Outcomes Using 2PIE

The model of exploitative and apparent competition between parasites, 2PIE, makes predictions that can help to contextualise and interpret previous experiments (Figures 7, 8, 9). To illustrate, we envision how 2PIEi and 2PIEc could produce these various outcomes, backing‐out a consistent mechanism *post hoc*. Thus, 2PIE models offer a way to potentially resolve otherwise seemingly inconsistent experimental outcomes. Then, it can guide the creation of new coinfection models tested in future experiments.

### 6.1 Coinfected or Not? What Within‐Host Factors Facilitate or Inhibit Coinfection?

#### 6.1.1 Priority Effects

*Theory*: Stronger inter‐ than intra‐specific competition ensures priority effects (via net positive feedback) where the parasite with a sufficiently high initial dose wins (Figure 7A,B; Table 1B).

*Empirical evidence*: In a rare, unequivocal demonstration of priority effects, the arrival order of strains of a bacterium ( *B. burgdorferi* ) determined which excluded the other within a host mouse (Figure 7B). The immune response likely did not explain priority effects (Devevey et al. 2015), so future work can pinpoint mechanisms (e.g., resource competition, interference, etc.) facilitating them (Figure 2B; Table 1B).

*Guiding future experiments*: Varying initial parasite dose (dose‐dependent assays) provides one possible way to delineate coinfection vs. priority effects. When parasites coinfect, different initial doses (e.g., different *P*_*2*_ and constant *P*_*1*_) do not change the qualitative long‐term outcome. With priority effects, sufficiently high initial density determines the winner (Table 1B). Such an experiment would reveal whether competitive exclusion or priority effects led to single infection. Even better, measurement of key quantities involved in *sensitivity* and *impact* ratios can delineate mechanisms that generate positive feedback.

#### 6.1.2 Competitive Exclusion

*Theory*: Differences in parasite traits can separate exclusion from coinfection. For example, for a given nutrient supply to hosts, a parasite with a higher feeding rate (all else equal) can exclude its competitor (Figure 7A,C). In contrast, one with a lower feeding rate may successfully coinfect.

*Empirical evidence*: Mice infected with malarial strain *P*_*1*_ or *P*_*3*_ (blue) were allowed to compete pairwise against a common malarial strain *P*_*2*_ (purple; Figure 7C; De Roode et al. 2005). In the *P*_*3*_—*P*_*2*_ pairing, regardless of order or arrival or delay between infections, *P*_*3*_ competitively excluded *P*_*2*_.

*Guiding future experiments*: Higher competitive ability of *P*_*3*_ may reflect a higher feeding rate on host energy. In contrast, if strain *P*_*1*_ had a lower feeding rate (hence less competitive), it could stably coinfect with *P*_*2*_. Such hypotheses about resource acquisition traits could await future tests (Table 1B).

### 6.2 Coinfection Community Structure

*Theory*: When parasites coinfect, nutrient supply to hosts (*S*) can mediate densities of the within‐host energy and immunity, and hence relative and absolute densities of parasites (Figure 8A). Thus, nutrient supply can shift ‘community structure’ of co‐infecting parasites within hosts. For instance, increasing nutrient supply favours the more immune‐resistant *P*_2_ over the less resistant *P*_*1*_.

*Empirical evidence*: This prediction could explain shifts in community structure of parasites in two different systems (Figure 8B). The first arose with mice infected by two species of gastrointestinal worms (case I; Budischak et al. 2015); the second used mosquito larvae infected by a microsporidian and protozoan parasite (case II; Fellous and Koella 2009). Both demonstrate how changing nutrient supply (*S*) favours one species (purple; positive slope) over the other (blue; negative slope; Figure 8B). In both cases, the relative abundance of coinfecting species can shift (or not) depending on *S*, too. However, future trait measurements would need to establish that higher *S* favoured the more resistant parasite, as 2PIE predicts (Table 1C).

*Guiding future experiments*: Much may be learned about coinfection and nutrient supply with a focus on individual hosts (reviewed in Ezenwa 2021). Hosts can exhibit high intra‐ and inter‐individual variation in infection outcomes (Merrill and Cáceres 2018). To illustrate, parasite densities in the mosquito case ranged among all individuals from coinfection to single infection via exclusion/priority effects to no infection (see reference to shaded regions in Figure 8A *v* 8C; replotting of Fellous and Koella 2009, detailed in Appendix C). Such variation could arise from individual differences in resource acquisition. If so, individuals nominally fed the same amount may fall functionally along different supply points (like in Figure 8A). Individuals consuming fewer resources would favour the less immune‐resistant parasite (*P*_*1*_) while those eating much more would favour the more resistant one (*P*_*2*_)—yielding exclusion in both cases (Figure 8A,C). The remaining hosts became coinfected, with parasites reaching different densities among hosts. Future experiments can test for the full range of possible infection outcomes at the individual scale, say, along broader nutrient gradients (Table 1C).

Furthermore, niche‐based insights from the within‐host level can be extended to the population scale, linking within‐ to between‐host dynamics. For instance, fluctuations in nutrient supply to hosts could shift competitive outcomes within hosts; that shift could then alter multi‐parasite outbreaks at the population (Hite and Cressler 2018; Ezenwa and Jolles 2011) and, potentially, ecosystem scale (Kendig et al. 2020).

### 6.3 Coinfection Burden

Finally, some hosts present a higher coinfection burden (density) than others, hinting at variation in intrinsic host resistance via clearance. How does variation in immune response govern coinfection burden?

*Theory*: Baseline allocation of energy to immunity (in 2PIEc via *a*_*b*_) squeezes parameter space for coinfection and reduces parasite burden (relative to 2PIEi: Figure 9A,B). Reduced burden allows the infected host to retain more energy for other metabolic work (Figure 9C,D). At lower nutrient supply (*S*), the ‘no infection’ outcome shifts from nutritional (*E*, yellow; Fig, 9A) to combined immune and nutritional clearance (*E‐I*, green; Fig, 9B). The 2PIEc model also predicts that increasing baseline allocation favours more immune‐resistant *P*_*2*_ over *P*_*1*_, eventually leading to *P*_*1*_'s exclusion (Figure 9E).

*Empirical evidence*: Consistent with these predictions, plant hosts treated with immune‐signalling hormone experienced lower prevalence of one of its fungal parasites, increased burden of infection by a competing fungus, and fewer coinfections (Halliday et al. 2018). Thus, hosts with higher allocation to immunity can resist infection more than those with lower allocation, leading to variation among hosts in infection burden (all else equal). Similarly, one clone of *Plasmodium chabaudi* showed reduced success in competition within immunocompetent mice (~*a*_*b*_ > 0; reconstituted with T‐cells) compared to immune‐deficient mice (*a*_*b*_ = 0; nude mice lacking T‐cells; Råberg et al. 2006). This result also echoed predicted outcomes of immune‐mediated competition (but *see* Barclay et al. 2008 for a counter‐example in the same system).

*Guiding future experiments*: First, one could test such predictions using strains with immune suppression of specific genes or metabolites involved in constitutive immunity (Chen et al. 2005). That could shift competition from a 2PIEc framework (with *a*_*b*_ > 0) to a more 2PIEi‐like one (with *a*_*b*_ = 0 at the extreme). Another test could involve measuring competitive outcomes in host genotypes differing in allocation to baseline immunity (Fuess et al. 2021; Table 1D).

## 7 Conclusion

Why do divergent infection dynamics arise within a host? Despite two decades of empirical research on within‐host infection dynamics, a mechanistic framework to explain coinfection and its diverse consequences has yet to emerge. Working toward that goal, we illustrate general principles for within‐host parasite interactions (via exploitative and immune‐mediated apparent competition) using a ‘2PIE’ model (Figures 3, 4, 5, 6). Although specifics will vary, these principles should apply to other within‐host niche models involving, say, two energy or immune sources (Figure 2). In the 2PIE example, we delineate how the interplay of three quantities – minimum resource requirements of (i) parasites for energy (*E*_j_*, akin to R*), and of (ii) the product of energy and parasites (*EP*_j_*) for immune cells, and (iii) maximal immune cells supported by each parasite (*I*_j_*) – provides a start for assembly of coinfection (Figures 4 and 5). Our work then establishes foundational principles for understanding mechanisms of parasite coinfection. In this model, coinfection requires a competition‐resistance trade‐off and that each parasite exerts a greater impact on its more sensitive niche factor. This arrangement produces stabilising, negative feedback. With priority effects, each parasite more strongly impacts the factor to which its competitor is most sensitive, producing positive feedback. Similar principles should apply to other mechanisms of within‐host competition between parasites.

We then synthesised how a niche competition framework can provide reasonable but *post hoc* explanations of some experimental results (Figures 7, 8, 9). First, it explains two ways to hinder coinfection: priority effects can favour early invaders, while competitive exclusion always inhibits one parasite (arising via e.g., fast feeders at low nutrient supply; Figure 7). Second, increasing nutrient supply in the coinfection region can favour the more immune resistant over the less resistant parasite, shifting community structure (Figure 8). Finally, greater investment in constitutive immunity squeezes opportunity (parameter space) for coinfection and reduces parasite burden, freeing up energy for the metabolic needs of hosts (Figure 9). Such insights will hopefully guide future experiments. However, this synthesis also underscores the need for experiments that can a priori predict within‐host infection outcomes with parameterised trait‐based models (Table 1).

Unpacking such within‐host mechanisms can improve our understanding of individual health. For instance, deworming trials of hosts coinfected with malaria and gastrointestinal worms show that increased availability of RBCs allows malaria to proliferate within the host, ultimately making them sicker (Budischak et al. 2018). Disentangling the mechanisms can then allow for correct plans of treatment to improve individual health (e.g., malarial drugs or vaccines followed by deworming). Together, our work synthesises within‐host niche‐based frameworks, providing a theoretical foundation for understanding competition outcomes. By applying model predictions, we contextualised key findings from two decades of research. Future models could capture more complexity of within‐host interactions. For instance, cooperation in the production of shared resources and interference (spite) can produce more contingency in coinfection outcomes (Sofonea et al. 2017). Additionally, most coinfection work focuses on biomedical applications. Other systems offer opportunities but require funding to advance both basic and applied research (Duffy et al. 2021; Bolker 2012). In the meantime, we aim here to catalyse a new combination of niche modelling and experimentation. This wave could help to mitigate severity and comorbidities of coinfections but also advance the development of preventive drug therapeutics and vaccines, ultimately enhancing individual health.

**TABLE 1:**
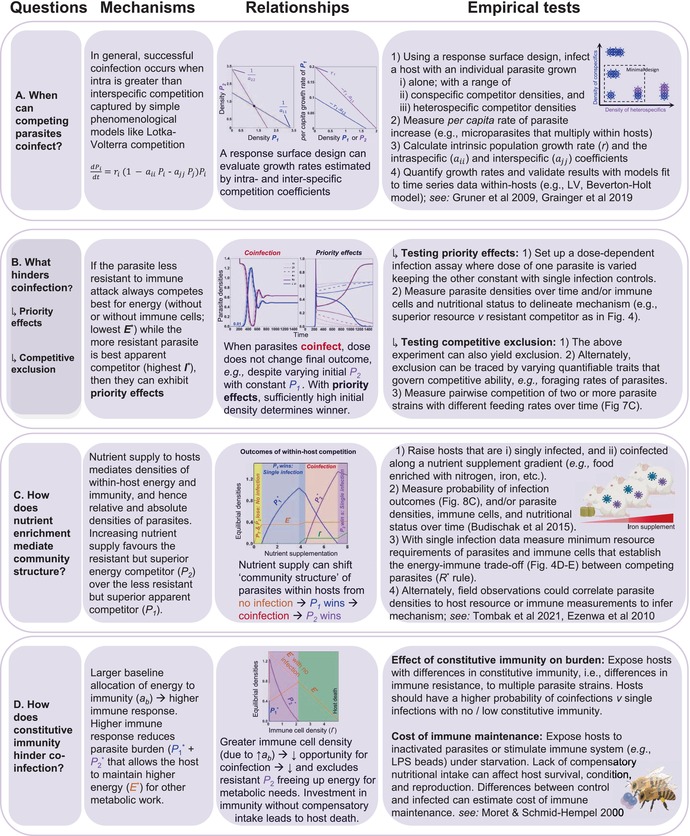
A guide to future coinfection experiments: The outcomes of within‐host competition can be tested by (A) measuring species' invasion growth rates via phenomenological models (e.g., Lotka‐Volterra), or (B–D) via parameterising a mechanistic model like those outlined in Figures 2, 3, 4, 5, 6, 7.

*Note:* ‘Questions’ outline fundamental queries surrounding causes and consequences of divergent infection outcomes. ‘Mechanisms’ summarise the theory, and ‘Relationships’ shows equations or correlations that can connect theory to experiments. ‘Empirical tests’ provides examples of how those relationships can be tested across a range of systems. Figure created using BioRender.com.

## Author Contributions

A.R. (lead) and S.R.H. conceptualised the paper. A.R. performed the analyses (lead), collated and synthesized the data, and wrote the first draft of the manuscript. S.R.H performed analysis (support) and edited the manuscript. Both authors substantially contributed to final revisions.

## Conflicts of Interest

The authors declare no conflicts of interest.

## Peer Review

The peer review history for this article is available at https://www.webofscience.com/api/gateway/wos/peer‐review/10.1111/ele.70104.

## Supporting information


Data S1.

## Data Availability

The code to reproduce all the analysis is available at https://doi.org/10.5281/zenodo.14996192.
