# Supplementary material for: On the Cause and Consequences of Coinfection: A General Mechanistic Framework of Within‐Host Parasite Competition
Source: Ecol Lett. 2025 Jun 27;28(6):e70104. doi: 10.1111/ele.70104 (PMC12203456; doi:10.1111/ele.70104)

On the cause and consequences of coinfection:
A general mechanistic framework of within-host parasite competition

Ashwini Ramesh[1,2*] and Spencer R Hall[2]

SUPPLEMENTAL APPENDIX

A. Feedback analysis in 2PIE

The two parasite-immune cell-energy model (2PIE; eq. 2, Table S1) represents within-host competition among parasites. Consider a case where two competing parasites (P_j) steal energy (E) from hosts and immune cells (I) that kill them (2PIE; Fig. 2E; Table S1). Hence, these parasites engage in simultaneous exploitative and immune-mediated apparent competition. To illustrate, we focus on one common variant of the immune activation where parasites induce production of immune cells (2PIEi, induced immunity).

Interior equilibrium

The 2PIEi model (eq. 2, Table S1) produces a single 'interior' (co) equilibrium (i.e., one with positive densities of the two niche factors, E and I, and the two parasites, P_1 and P_2, respectively):

$$E^*_{co} = \frac{E_1^* E_2^* \left(f_{IP_1} - f_{IP_2}\right)}{f_{IP_1}E_1^* - f_{IP_2}E_2^*} \qquad \text{(A1.a)}$$

$$I^*_{co} = \frac{m_P\left(E_2^* - E_1^*\right)}{f_{IP_1}E_1^* - f_{IP_2}E_2^*} \qquad \text{(A1.b)}$$

$$P^*_{1,co} = P_{1-I}\left(\frac{T_1 - f(S)}{f_2(I)EP_2^* - f_1(I)EP_1^*}\right) \qquad \text{(A1.c)}$$

$$P^*_{2,co} = P_{2-I}\left(\frac{f(S) - T_2}{f_2(I)EP_2^* - f_1(I)EP_1^*}\right) \qquad \text{(A1.d)}$$

where the equilibrial expressions (eq. A1.a-d) involve minimal energy requirements for each parasites j without immune attack, E_j^*, and minimal energy x parasite j density for the immune system, EP_j^*:

minimal requirement for energy without immunity: $E_j^* = \frac{e_{P_jE}\, m_P}{f_{P_j}}$ (A2.a)

minimal EP requirement for the immune system: $EP_j^* = \frac{e_I m_I}{e_{IP_j} f_{IP_j}}$ (A2.b)

and per capita 'consumption' of energy by parasite j: $f_j(I) = f_{P_j} + e_{IP_j} f_{IP_j} I^*_{co}$ (A2.c)

where $f_j(I)$ captures the per capita sum of direct consumption of energy by parasites (at rate f_{Pj}) and of that consumed following immune attack ($e_{IP_j} f_{IP_j}\, I^*_{co}$; eq. A2.c); and finally, where 'transcritical' thresholds (T_j) and minimal parasite densities needed for immune activation, $P_{j\text{-}I}$, given E_{co}^*, are:

T_1: $rE^*_{co} + f_2(I)EP_2^*$ (A3.a)

T_2: $rE^*_{co} + f_1(I)EP_1^*$ (A3.b)

$P_{j-I} = EP_2^* / E_{co}^*$ (A3.c)

respectively. A 'feasible' equilibrium (with positive densities) first requires a competition-resistance trade-off (Fig. 4C). If P_1 is less resistant ($f_{IP_1} > f_{IP_2}$; for $E_{co}^* > 0$, eq. A1.a) but the superior energy competitor without immune attack ($E_2^* > E_1^*$ for $I_{co}^* > 0$, eq. A1.b), the trade-off must be strong enough to satisfy $f_{IP_1}E_1^* > f_{IP_2}E_2^*$. As shown below, these conditions ensure parasite nullclines intersect (and involve feedback coming from the sensitivity submatrix, **S**). Then, positive density of parasites requires both numerator and denominator to have the same sign (and as shown below, this sign has implications for stability and the slope of E and I nullclines in P_1-P_2 space; Fig. 4E). For now: if the denominators are both positive ($f_2(I)EP_2^* > f_1(I)EP_1^*$), then positive P_1 and P_2 require that assimilated energy, $f(S)$, falls intermediately, i.e., that $T_2 < f(S) < T_1$ (as shown below, this case leads to co-infection; eqs. A1.c,d, A3.a,b). If the denominators are both negative, feasibility requires $T_2 > f(S) > T_1$ (leading to priority effects).

The Jacobian matrix, feedback loops, and key ratios:

A Jacobian matrix characterizes the direct effects of a species or niche factor on the growth rate of another. More specifically, if growth rate (production) of X is $dX/dt = G_X$, then the direct effect of an increase in density of Y on X's growth rate is $J_{XY} = \partial G_X / \partial Y$. The Jacobian matrix for 2PIE at the feasible interior equilibrium (eq. A1-3), $\mathbf{J_{2PIEi}}$, in order of E, I, P_1, P_2 is:

$$\mathbf{J_{2PIEi}} = \begin{bmatrix} J_{EE} & 0 & J_{EP_1} & J_{EP_2} \\ J_{IE} & 0 & J_{IP_1} & J_{IP_2} \\ J_{P_1E} & J_{P_1I} & 0 & 0 \\ J_{P_2E} & J_{P_2\mathrm{E}} & 0 & 0 \end{bmatrix} =$$

$$\begin{bmatrix} J_{EE} & 0 & -f_{P_1}E^* & -f_{P_2}E^* \\ \left(\sum e_{IP_j} f_{IP_j} P_j^*\right) E^* & 0 & e_{IP_1} f_{IP_1} I^* E^* & e_{IP_2} f_{IP_2} I^* E^* \\ \frac{f_{P_1}P_1^*}{e_{P_1E}} & -f_{IP_1}P_1^* & 0 & 0 \\ \frac{f_{P_2}P_2^*}{e_{P_2E}} & -f_{IP_2}P_2^* & 0 & 0 \end{bmatrix}$$

(A4)

where the Jacobian elements are evaluated at the interior equilibrium (but 'co' subscripts are dropped for niche factors energy, E^* and immune cells, I^*, and the two competing parasites, P_1^* and P_2^*). That matrix can be compartmentalized as:

$$\mathbf{J_{2PIEi}} = \begin{bmatrix} \mathbf{L} & \mathbf{I} \\ \mathbf{S} & \mathbf{C} \end{bmatrix} \quad \text{(A5)}$$

with submatrices for limiting niche factors (**L**), impacts of parasites on those niche factors (**I**), sensitivity of parasite growth to niche factor (**S**), and direct competition between parasites (**C**):

$$\mathbf{L} = \begin{bmatrix} J_{EE} & 0 \\ J_{IE} & 0 \end{bmatrix}, \mathbf{I} = \begin{bmatrix} J_{EP_1} & J_{EP_2} \\ J_{IP_1} & J_{IP_2} \end{bmatrix}, \mathbf{S} = \begin{bmatrix} J_{P_1E} & J_{P_1I} \\ J_{P_2E} & J_{P_2I} \end{bmatrix}, \mathbf{C} = \begin{bmatrix} 0 & 0 \\ 0 & 0 \end{bmatrix}. \quad \text{(A6)}$$

This matrix of direct effects (eqs. A4-6) provides the information needed to characterize stability of the interior equilibrium (eqs. A1-3), ultimately determining if it produces coinfection or priority effects. More specifically, its stability depends on the sign of feedback looped through all four interacting species and niche factors (level 4 feedback, F_4; as shown previously, feedback looped through 1-3 interactors all are negative [Ramesh & Hall 2023]). At this level, coinfection requires negative feedback, $F_4 < 0$, at a feasible interior. This F_4 is the determinant of the Jacobian (F_4 = -det[$\mathbf{J_{2PIEi}}$]). It can be written three different ways, each contributing unique biological interpretation:

$$F_4 = \underbrace{\underbrace{J_{IP_1}J_{P_2I}J_{EP_2}J_{P_1E}}_{(i)P_1\text{ fuels immune cells}} + \underbrace{J_{EP_1}J_{P_2E}J_{IP_2}J_{P_1I}}_{(ii)P_1\text{starves immune cells}}}_{\textbf{Inter}-\text{specific competition}} \underbrace{- \underbrace{J_{IP_1}J_{P_1I}J_{EP_2}J_{P_2E}}_{(iii)P_1\text{ is attacked; }P_2\text{ eats}} - \underbrace{J_{EP_1}J_{P_1E}J_{IP_2}J_{P_2I}}_{(iv)P_2\text{ is attacked; }P_1\text{ eats}}}_{\textbf{Intra}-\text{specific competition}} \quad \text{(A7.a)}$$

$$F_4 = -\det[\boldsymbol{L}\,\boldsymbol{C} - \boldsymbol{I}\,\boldsymbol{S}] = -\det[\boldsymbol{I}]\det[\boldsymbol{S}] = -(J_{EP_1}J_{IP_2} - J_{EP_2}J_{IP_1})(J_{P_1E}J_{P_2I} - J_{P_2E}J_{P_1I}) \quad \text{(A7.b)}$$

$$F_4 = -J_{P_1E}\,J_{P_2E}\,J_{EP_1}\,J_{EP_2} \;\; x \;\; \underbrace{\left(\frac{J_{P_1I}}{J_{P_1E}} - \frac{J_{P_2I}}{J_{P_2E}}\right)}_{\text{sensitivity ratios }(\alpha_1-\alpha_2)} \;\; x \;\; \underbrace{\left(\frac{J_{IP_1}}{J_{EP_1}} - \frac{J_{IP_2}}{J_{EP_2}}\right)}_{\text{impact ratios }(\varepsilon_1-\varepsilon_2)}. \quad \text{(A7.c)}$$

In the first way (eq. A7.a), F_4 is written as the sum of two positive (destabilizing) and two negative (stabilizing) loops (Fig. 6.A). The positive loops (i-ii) are competitive interactions via the other parasite species or *inter*-specific competition, while the negative loops (iii-iv) are competitive interactions of each species on itself via host energy or immune attack or *intra*specific competition. Then, the relative strength of intra- vs inter-specific competition governs whether parasites coexist or show priority effects. When relative effects of intraspecific competition are stronger, coexistence ensues (due to net negative feedback); when interspecific competition dominates, priority effects emerge instead (net positive feedback).

In the second way (eq. A7.b), we use a formula for the determinant of block matrices made of **L**, **C**, **I**, and **S** (eqs. A5,6). Since **C** = 0, feedback only depends upon the feedback of the sensitivity, -det(**S**), and the impacts, -det(**I**), submatrices. Feedback at level 4 is negative if both **S** and **I** submatrices produce negative feedback - or if both determinants of **S** and **I** are positive (as pursued below). With some algebra, we can simplify det(**S**) and det (**I**) to proportional ($\propto$) quantities imbued with meaning:

$$\det(\mathbf{S}) = J_{1E}\,J_{2I} - J_{1I}\,J_{2E} \propto f_{IP_1}E_1^* - f_{IP_2}E_2^* \quad \text{(A8.a)}$$

$$\det(\mathbf{I}) = J_{P_1E}J_{P_2I} - J_{P_2E}J_{P_1I} \propto f_2(I)EP_2^* - f_1(I)EP_1^* \quad \text{(A8.b)}$$

which are the denominators of the niche equilibria (E_{co}^*, I_{co}^*; eqs. A1.a,b) and that of the parasites ($P_{co,1}^*$ and $P_{co,2}^*$; eqs. A1.c,d), respectively. These two determinants have two more related interpretations for each. The sensitivity of P_j's fitness to host energy, $\partial r_j/\partial E$, is proportional to $1/E_j^*$; similarly, sensitivity of its fitness to immune cells, $\partial r_j/\partial I$, is $-f_{IP_j}$. Hence, det(**S**) is proportional to the difference in the ratio of sensitivities of each parasite to immune cells / energy (eq. A8.a). Similarly, the impact of each parasite j on

E (J_{EP_j}) is $f_j(I)$, while the impact of each on I (J_{IP_j}) is proportional to $1/EP_j^*$. Hence, with algebra, det(**I**) is proportional to the difference in ratio of impacts of parasites on immune cells/energy ($[f_j(I)EP_j^*]^{-1}$; equ. A8.b). More interpretation of these sensitivities and impacts follow.

Finally, in the third variation, that four-interactor feedback can be reorganized as proportional to (a)symmetries of two quantities (eq. A7.c). Those quantities reflect ratios of how each parasite is *sensitive* (α_j) and has *impacts* on (ε_j) their immune cells and energy (Fig. 6B). The *sensitivity* ratio for each parasite P_j is the ratio of the effects of the immune cells and energy on the competitor (J_{P_jI} and J_{P_jE}, respectively: eq. A7.c) and can be represented proportionally to key traits that govern them (Fig. 6D):

$$\alpha_j = J_{P_jI}/J_{P_jE} \propto \frac{e_{P_jE} f_{IP_j}}{f_{P_j}}. \quad \text{(A9)}$$

Additionally, the *impacts* ratio for each P_j, is the ratio of the effects of P_j on its immune cells (J_{IP_j}) vs. on its energy (J_{EP_j}; A7.c) and can be represented proportionally to key traits that govern them:

$$\varepsilon_j = J_{IP_j}/J_{EP_j} \propto \frac{e_{IP_j} f_{IP_j}}{f_j(I)}. \quad \text{(A10)}$$

This trait ratio is like an 'impact vector' *sensu* Leibold (1996). Stability hinges on (a)symmetry of these ratios (and the traits and minimal requirements proportional to them). The remainder of this appendix will focus on these key ratios governing trade-offs of traits and its deep links to nullclines and assembly rules.

To forge connections between the sensitivity and impact ratios, nullclines, and assembly rules, we represent these differences in ratios as differences in traits (as in Fig. 6D):

$$\underbrace{\left(\frac{e_{P_1E} f_{IP_1}}{f_{P_1}} - \frac{e_{P_2E} f_{IP_2}}{f_{P_2}}\right)}_{sensitivity\ \text{"traits"}} \; x \; \underbrace{I_{int}^* \left(\frac{e_{IP_1} f_{IP_1}}{f_1(I)} - \frac{e_{IP_2} f_{IP_2}}{f_2(I)}\right)}_{impact\ \text{"traits"}} \quad \text{(A11)}$$

and that can be rewritten, in parallel order, as proportional to differences in minimal quantities ('minima'):

$$\underbrace{(f_{IP_1}E_1^* - f_{IP_2}E_2^*)}_{sensitivity\ \text{"minima"}} \; x \; \underbrace{(f_2(I)EP_2^* - f_1(I)EP_1^*)}_{impact\ \text{"minima"}} \quad \text{(A12)}$$

(as in Fig. 6E), where quantities E_j^*, $EP_{I,j}^*$, and $f_j(I)$ follow above (eq. A2). The first difference (eq. A12; also eq. A8.a), i.e., the *sensitivity* "minima", is a statement about the slopes of each parasite's nullclines in E-I space – that is, the combination of E (influencing births) and I (deaths) that give zero fitness for the parasite. For parasite j, that nullcline is (Fig. 4A):

$$I = \underbrace{\left(\frac{m_P}{f_{IP_j}E_j^*}\right)}_{slope} E \; - \; \underbrace{\frac{m_P}{f_{IP_j}}}_{intercept} \quad \text{(A13)}$$

which has an E-axis intercept of E_j^* (eq. A2.a) and slope proportional to 1 / ($f_{IP_j}E_j^*$) or 1 / (*sensitivity* "minima" related to α_j; eq. A12). Any combination of E and I to the right of the nullcline falls within the fundamental niche of the parasite (Fig. 4A). Given the competition-resistance trade-off, then, the more vulnerable parasite with lower E_j^*, P_1, has a nullcline with shallower slope than P_2 (Fig. 4C). The two nullclines cross at E_{co}^* and P_{co}^* (eq. A1.a,b; Fig. 4C). Hence, that portion of the interior equilibrium is feasible, in part, because P_1 is more sensitive to immune attack and while P_2 is more sensitive to energy.

Feasibility of the interior equilibrium also requires that nullclines for energy and immune cells cross, too (eq. A1.c,d; Fig. 4E). These conditions involve both the ratio of impacts but also the transcritical thresholds (eq. A3). To explain these connections, we first describe the E and I nullclines as evaluated and their interior values, E_{co}^* and I_{co}^* (eq. A1.a,b):

E nullcline: $P_2 = P_{2\text{-}E} - (f_1[I_{co}^*] / f_2[I_{co}^*])\, P_1$ (A14.a)

with: P_2 axis intercept: $P_{2\text{-}E} = (f[S] - r\, E_{co}^*) / (f_2[I_{co}^*]\, E_{co}^*)$ (A14.b)

P_1 axis intercept: $P_{1\text{-}E} = (f[S] - r\, E_{co}^*) / (f_1[I_{co}^*]\, E_{co}^*)$ (A14.c)

where the slope is the ratio of per capita 'consumption' terms (eq. A2.c). The nullcline for E (A14.a) is the combination of P_1 and P_2 that 'consumes' all net production of energy, $f(S) - rE_{co}^*$ (by definition). All densities of parasites below this line lead to growth of energy within hosts (i.e., energy is under-consumed, so dE/dt > 0; yellow region, Fig. 4B). Hence, the P_2 axis intercept is the density of P_2, $P_{2\text{-}E}$, that ensures all production is consumed (eq. A14.b), given per capita 'consumption' rate $f_2[I_{co}^*]\, E_{co}^*$. Similarly, the P_1 axis intercept denotes the density of P_1, $P_{1\text{-}E}$, that consumes all production (A14.c). Both intercepts increase with

S – with more resource supply, the host can support more parasites when the niche environment is set at the interior (E_{co}^* and I_{co}^*; eq. A1.a,b).

Then, the immune nullcline at interior E_{co}^* is (Fig. 4B,E):

I nullcline: $P_2 = P_{2\text{-}I} - (EP_2^* / EP_1^*)\, P_1$ (A15.a)

with: P_2 axis intercept: $P_{2\text{-}I} = EP_2^* / E_{co}^*$ (A15.b)

P_1 axis intercept: $P_{1\text{-}I} = EP_1^* / E_{co}^*$. (A15.c)

The nullcline for I (A15.a) is the combination of P_1 and P_2 that meets the minimal needs of the immune system for activation (and positive immune density is required for coinfection). All densities of parasites above the line increase growth of immune cells ($dI/dt > 0$; green region, Fig. 4B). The P_2 axis intercept is the density of P_2, $P_{2\text{-}I}$, that supports immune activation (at EP_2^*), given E_{co}^* (eq. A15.b; see also eq. A3.c). Similarly, the P_1-axis intercept $P_{1\text{-}I}$ is its density that would support immune activation alone (at E_{co}^*; eq. A15.c).

Stability and coinfection require a symmetry of difference in sensitivity (eq. A9) and impact (eq. A10) ratios. If P_1 is the superior energy competitor without immune cells ($E_1^* < E_2^*$) but P_2 better resists immune attack ($f_{IP_1} > f_{IP_2}$), then $\alpha_1 - \alpha_2 > 0$ (given eq. A9). That difference in sensitivity ratios is positive because P_1 is relatively more sensitive to immune cells while P_2 is more to energy (see also eq. A1.a,b). Additionally, as described above (eq. A13), each P_j's nullcline slope is proportional to $1 / (f_{IP_j} E_j^*)$. Hence, $\alpha_1 > \alpha_2$ means that P_1's nullcline is flatter (i.e., relatively more sensitive to I) than P_2's, ensuring that nullclines of both parasites cross in E-I space. Then, if P_1 has larger impact on immune cells and P_2 has larger on energy, the $\varepsilon_1 > \varepsilon_2$, hence $f_2(I)EP_2^* - f_1(I)EP_1^* > 0$ (eqs. A8.b, A12). This geometry means that that I nullcline's slope, $-EP_2^* / EP_1$ (eq. A15.a) is more steeply (negatively) sloped than the E nullcline, $-f_1(I_{int}^*) / f_2(I_{int}^*)$ (eq. A14.a). That relationship (symmetry: $\alpha_1 > \alpha_2$ and $\varepsilon_1 > \varepsilon_2$) sets the stage for coinfection. In contrast, priority effects arise if $\varepsilon_1 < \varepsilon_2$, i.e., if there is an asymmetry in the difference in sensitivity and impact ratios ($\alpha_1 > \alpha_2$ still but now $\varepsilon_1 < \varepsilon_2$). In this case, E's nullcline is steeper because P_1 has a larger impact on it (while P_2 has large impact on immune cells).

Either coinfection or priority effects requires that E and I nullclines cross interiorly (in positive P_1 and P_2 space). Specifically, coinfection requires that parasite null clines cross, niche nullclines cross, and this crossing occurs where all variables are strictly non-negative. The relative positions of their intercepts ensures that nullclines cross; however, the order in which they cross with increasing S determines the outcome. For coinfection, right at the low-S border of the coinfection region, the host niche produces (supports) just enough P_1, $P_{1\text{-}E}$, to meet the minimal requirement for immune activation at the interior, $P_{1\text{-}E}$, i.e., $P_{1\text{-}I} = P_{1\text{-}E}$ (and $f(S) = T_2$; from eqs. A13.c, A14.c) when energy and immune density sit at their interior values (E_{co}^* and I_{co}^*). Here, the P_1 axis intercepts intersect. The host niche would not support enough P_2 to do so ($P_{2\text{-}I} > P_{2\text{-}E}$; P_2 is in 'deficit'). As S increases, intercepts of the E nullcline push up ($P_{2\text{-}E}$) and right ($P_{1\text{-}E}$). In this region, the interior equilibrium becomes feasible, resource supply sits between the two transcritical boundaries ($T_2 < f(S) < T_1$; eq. A3.a,b), the E-P_1-I equilibrium becomes invasible by P_2, and the host niche environment would support P_1 in excess of I's activation requirement (a surplus; Fig. 4E). As the community of parasites becomes more resistant overall, shifting to less P_1 and more P_2, that surplus is eliminated. All net production, $f(S) - r\,E_{co}^*$, is consumed while both parasites maintain immune activation, at E_{co}^* ($P_{co,1}^* + P_{co,2}^*$; Fig. 4E). Then, at high enough S (at $f(S) = T_1$), more resistant P_2 could support the immune system itself, so $P_{2\text{-}I} = P_{2\text{-}E}$ (i.e., the deficit of P_2 has been eliminated). At this upper transcritical, less resistant P_1 is pushed to zero – it is competitively excluded. At any higher S, P_2 wins via resource competition, and its E-P_2-I boundary equilibrium is not invasible by P_1.

With priority effects, the order of crossing of nullclines is reversed. At the start of the priority effects region, the host environment provides just enough of resistant P_2 to maintain immune activation at the interior ($P_{2\text{-}I} = P_{2\text{-}E}$, at $f(S) = T_1$) while P_1 sits in deficit ($P_{1\text{-}I} > P_{1\text{-}E}$). With further increases in S, the surplus of P_2 and the deficit of P_1 combine to consume all production while supporting immune activation. However, P_1 cannot invade an E-P_2-I boundary equilibrium. At high enough S (at $f(S) = T_2$), the deficit of P_1 would be eliminated ($P_{1\text{-}I} = P_{1\text{-}E}$). At any S above this point, resistant P_2 competitively displaces P_1 via apparent competition.

Appendix Table S1. Variables and parameters, their definition (with units), and corresponding default values used in analysis of the 2PIE models (Eq. 1; in Figs. 3 - 9).

Var	Definition	Values Fig. 3-9[a,b]
I	Immune cell density (L^{-1})	
P_j	Parasite j density (L^{-1})	
E	Host energy density (L^{-1})	
e_{IP_j}	Induced energy allocation rate per parasite j killed by the immune response (L)	3, 4.7
f_{IP_j}	Killing rate, immune cell on parasite j ($L \cdot day^{-1}$)	0.9, 0.4
m_I	Immune cells background mortality rate (day^{-1})	0.4
e_I	Cost of producing immune cell	2.5
e_{P_1E}	Cost of producing parasite 1	3.55, 4[c]
e_{P_2E}	Cost of producing parasite 2	3.4
f_{P_j}	Feeding rate per parasite j on energy ($L \cdot day^{-1}$)	var[b],7.2, 10.63
m_P	Parasite background mortality rate (day^{-1})	0.8
f_E	Host resource assimilation rate ($L^{-1} \cdot day^{-1}$)	7.8
S	Food supply point, hosts (L^{-1})	var[a,b]
h	Resource ingestion half-saturation constant (L^{-1})	3.2
r	Rate of host energy use for non-parasite purposes (day^{-1})	3.7
a_b	Baseline energy allocation to immunity (day^{-1})	2PIEi: 0<br>2PIEc: 0.1

[a] Values chosen to ensure P_1 is the superior competitor for resources without enemies (parasite: $E_1^* < E_2^*$) while P_2 is more resistant to attack to enemies ($f_{IP_1} > f_{IP_2}$). Additionally, they guarantee the *sensitivity* ratio has structure $\alpha_1 > \alpha_2$. [b] See values on figures and/or captions. [c] e_{P_1E} only in Fig. 9

Appendix Table S2. Summary of the outcomes of competition of two species competing for a shared energy while attacked by an immune system in the 2PIEi/2PIEc models (Eq. 1; Table S1).

**Condition / criterion**	**Coinfection**	**Priority Effects**
Four species feedback, F_4 [a]	$F_4 < 0$<br>(net negative feedback)	$F_4 > 0$<br>(net positive feedback)
Sensitivity ratio ranking [a]	$\alpha_1 > \alpha_2$<br>competition ($E_1^* > E_2^*$) – resistance ($f_{IP_1} > f_{IP_2}$) trade-off<br>P_1 more sensitive to I<br>P_2 more sensitive to E	$\alpha_1 > \alpha_2$ (same)<br>same competition-resistance trade-off
Impacts ratio ranking [b]	$\varepsilon_1 > \varepsilon_2$<br>$P_1$ more strongly impacts I,<br>P_2 more strongly impacts E	$\varepsilon_1 < \varepsilon_2$<br>flip in impact strengths among parasites
Ranking of ratios [a,c]	symmetrical	asymmetrical
Superior resource competitor [d]	P_2<br>($E_2^* < E_{1,I}^*$ or $E_{2,I}^* < E_{1,I}^*$)	P_1<br>($E_{1,I}^* < E_{2,I}^*$)
Superior apparent competitor [d]	P_1<br>($I_1^* > I_2^*$)	P_2<br>($I_2^* > I_1^*$)
Smaller transcritical? [e]	T_2<br>$T_2 < f(S) < T_1$	T_1<br>$T_1 < f(S) < T_2$
At lower S, P_1 wins via[f]	apparent competition<br>(higher I^*)	resource competition<br>(lower E^*)
At higher S, P_2 wins via[f]	resource competition<br>(lower E^*)	apparent competition<br>(higher I^*)
Switch in competitive rankings? [f]	yes	no
Stronger form of competition [g]	intra- > inter-specific	inter- > intra-specific

[a] Level 4 feedback (eq. A7). We assume that P_1 is the superior nutrient competitor ($E_1^* < E_2^*$) without immune cells, but that P_2 better resists immune attack ($f_{IP_1} > f_{IP_2}$). This *sensitivity* ranking enables a 'feasible interior' equilibrium (equ. A1). [b,c] Notice how the symmetry shifts because of change in the ranking of the *impact* ratios. [d] In region with a feasible interior equilibrium (eq. A1). See also Figs. 4, 5. [e] Coinfection also requires sufficiently intermediate resource supply, S, as consumed by hosts, $f(S)$ (falling in between the two transcriticals: $T_2 < f(S) < T_1$); priority effect requires $T_1 < f(S) < T_2$ (eq. A3; Figs. 3-5) [f] The superior resource competitor has the lower E^* (survives on lower energy, starving its competitor), but the superior apparent competitor has higher I^* (supports more immune cells, enhancing mortality on its competitor; Figs. 4,5). Just outside the coexistence or priority effects windows, competitive exclusions arises at lower and higher S for different reasons. The switch means the superior competitor without immune cells (P_1) becomes the inferior competitor with immune cells. [g] Interspecific competition (positive feedback) works through loops (i) and (ii) of F_4 [eq. A7.a; Fig. 6A]; intraspecific competition (negative feedback) works through loops (iii) and (iv). Stronger intraspecific competition leads to coinfection at a stable interior.

B. Some analysis of other niche models

The 2 Parasite – 2 Energy (2P2E) model aka the Resource Ratio Model

Here we outline the stability analysis for the coexistence *vs* priority effects for a within-host variation of the resource ratio model (Fig. 2C; Tilman 1982).

Growth rate of parasite P_j (eq. B1.a): The two parasites consume two within-host resources, E_i, at feeding rate ($f_{P_jE_i}$) and energy per parasite conversion, $e_{P_jE_i}{}^{-1}$. These resources are linearly substitutable. The parasites die at (shared) background rate m_P .

Growth rate of resources, E_i (eq. B1.b): The host consumes two resources, S_i, via Monod functions with maximal assimilation rate f_{E_i} and half-saturation constant h_i. Those external resources, converted to two internal resources (or energy) within the host (E_j), are lost at fixed rate r_i for use by hosts (for metabolic needs). Each within-host resources' net production, then, is $f_i(S) - r_i E$. Additionally, host resource is consumed by parasites. The 2P2E model is thus:

$$\frac{dP_j}{dt} = \left(\Sigma \frac{f_{P_jE_i}\ E_i}{e_{P_jE_i}} - m_P\right) P_j \quad \text{(B1.a)}$$

$$\frac{dE_i}{dt} = \frac{f_{E_i}S_i}{h_i+S_i} - r_i\, E_i - \left(\Sigma f_{P_jE_i}\, P_j\right) E_i\ . \quad \text{(B1.b)}$$

The Jacobian matrix, feedback loops, and key ratios:

First, the Jacobian matrix for 2P2E at its' feasible interior equilibrium, $\mathbf{J_{2P2E}}$, in order of E_1, E_2, P_1, P_2 is:

$$\mathbf{J_{2P2E}} = \begin{bmatrix} J_{E_1E_1} & 0 & J_{E_1P_1} & J_{E_1P_2} \\ 0 & J_{E_2E_2} & J_{E_2P_1} & J_{E_2P_2} \\ J_{P_1E_1} & J_{P_1E_2} & 0 & 0 \\ J_{P_2E_1} & J_{P_2E_2} & 0 & 0 \end{bmatrix} = \begin{bmatrix} \mathbf{L} & \mathbf{I} \\ \mathbf{S} & \mathbf{C} \end{bmatrix} \quad \text{(B2)}$$

where submatrices for limiting niche factors (**L**), impacts on niche (**I**), sensitivity to niche (**S**), and direct competition (**C**) are:

$$\mathbf{L} = \begin{bmatrix} J_{E_1E_2} & 0 \\ 0 & J_{E_2E_2} \end{bmatrix}, \mathbf{I} = \begin{bmatrix} J_{E_1P_1} & J_{E_1P_2} \\ J_{E_2P_1} & J_{E_2P_2} \end{bmatrix}, \mathbf{S} = \begin{bmatrix} J_{P_1E_1} & J_{P_1E_2} \\ J_{P_2E_1} & J_{P_2E_2} \end{bmatrix}, \mathbf{C} = \begin{bmatrix} 0 & 0 \\ 0 & 0 \end{bmatrix}. \quad \text{(B3)}$$

As seen above (eq. A7), the stability of the interior equilibrium - determining coinfection vs. priority effects - depends on the sign of level 4 feedback, F_4. Coinfection requires negative level 4 feedback, $F_4 < 0$. This level of feedback is the determinant of the Jacobian ($F_4 = -\det[\mathbf{J_{2P2E}}]$). It can be written in the three different ways, following the 2PIE script (eq. A7), each contributing biological interpretation of the feedback:

$$F_4 = \underbrace{\underbrace{J_{E_1P_1}J_{P_2E_1}J_{E_2P_2}J_{P_1E_2}}_{(i)P_1 \text{ starves } P_2\ via\ E_1} + \underbrace{J_{E_2P_1}J_{P_2E_2}J_{E_1P_2}J_{P_1E_1}}_{(ii)P_1 \text{ starves } P_2\ via\ E_2}}_{\textbf{Inter}\text{–specific competition}} - \underbrace{\underbrace{J_{E_1P_1}J_{P_1E_1}J_{E_2P_2}J_{P_2E_2}}_{(iii)P_1 \text{ eats } E_1;\ P_2 \text{ eats } E_2} - \underbrace{J_{E_1P_2}J_{P_2E_1}J_{E_2P_1}J_{P_1E_2}}_{(iv)P_2 \text{ eats } E_1;\ P_1 \text{ eats } E_2}}_{\textbf{Intra}\text{–specific competition}} \quad \text{(B4.a)}$$

$$F_4 = -\det[\mathbf{L\,C} - \mathbf{I\,S}] = -\det[\mathbf{I}]\det[\mathbf{S}] = -(J_{E_1P_1}J_{E_2P_2} - J_{E_1P_2}J_{E_2P_1})(J_{P_1E_1}J_{P_2E_2} - J_{P_1E_2}J_{P_2E_1}) \quad \text{(B4.b)}$$

$$F_4 = -J_{P_1E_1}J_{E_1P_1}J_{P_2E_2}J_{E_2P_2}\ \ x\ \ \underbrace{\left(\frac{J_{P_1E_2}}{J_{P_1E_1}} - \frac{J_{P_2E_2}}{J_{P_2E_1}}\right)}_{sensitivity\ ratio\ (\alpha_1 - \alpha_2)}\ \ x\ \ \underbrace{\left(\frac{J_{E_2P_1}}{J_{E_1P_1}} - \frac{J_{E_2P_2}}{J_{E_1P_2}}\right)}_{impacts\ ratio\ (\varepsilon_1 - \varepsilon_2)} \quad \text{(B4.c)}$$

In the first way (B4.a), F_4 is written as the sum of two positive (destabilizing) and two negative (stabilizing) loops. The positive loops (i-ii) are competitive interactions via the other parasite species or *inter*specific competition. For instance, P_1 consumes E_1 ($J_{E_1P_1}$) which depresses P_2 (via $J_{P_2E_1}$) which lowers its consumption E_2 ($J_{E_2P_2}$) which increases resources for P_1 (increased births, $J_{P_1E_2}$), competing the first loop with positive feedback. Meanwhile the negative loops (iii-iv) are competitive interactions of each species on itself via resource competition or *intra*specific competition. The strength of intra- vs inter-specific competition governing coexistence vs priority effects arises. When relative effects of intra- is greater than inter-specific competition then coexistence ensues; vice-versa for priority effects.

In the second way (B4.b), we use a formula for the determinant of block matrices. Feedback only depends upon the feedback of the sensitivity-to, -det(**S**), and of the impacts-on submatrix, -det(**I**), since **C** = 0. Feedback at level 4 is negative if both **S** and **I** submatrices produce negative feedback - or if both determinants of **S** and **I** are positive.

Finally, in the third variation (B4.c), that four-species loop product can be reorganized as the *sensitive*

(α_j) – *impacts* (ε_j) ratio on their two sources of energy. Here, the *sensitivity ratio* is the net effect of energy source 2 to energy source 1 on P_j, *i.e.,* increasing either energy density has a positive effect on P_j. Additionally, the *impacts* ratio for each P_j, is the ratio of the effects of P_j on energy source 2 *vs* 1. Then, the *impacts* ratio is the net effect of density of P_j on the growth rate of energy sources, *i.e.,* increasing parasite density has a negative effect on energy. Stability hinges on (a)symmetry of these ratios. Here, coinfection minimally requires each species to trade off their requirements for each energy resource. If each parasite has larger *impact* on the resource to which its fitness is most *sensitive*, it yields a symmetry of *sensitivity-impact* ratios ($\varepsilon_1 > \varepsilon_2$ and $\alpha_1 > \alpha_2$, respectively). Additionally, if resource supply and ratios are sufficiently intermediate (enabling single-species ['boundary'] equilibria to fall within each competitors niches), then coinfection at a stable equilibrium can arise. For priority effects, the competition trade-off did not change ($\alpha_1 > \alpha_2$) but the *impacts* ratios flip ($\varepsilon_1 < \varepsilon_2$). Hence, when each parasite has greatest impact on the niche dimension to which its competitor is most sensitive, net positive feedback and priority effects prevail.

The 2 Parasite – 2 Immune cells (2P2I) model aka the "Immune Ratio" model

Here we outline the stability analysis for the coexistence *vs* priority effects for an immune ratio model (first conceptualized here). Here, two parasites can be attacked by two different arms of the immune system (2P2I; Fig 2D but no interference between $I_1 - I_2$ i.e., the grey shading does not apply)

Growth rate of immune cells I (eq. B5.a)*:* In 2P2I, immune cells (I_i) proliferate after attack on parasite *j* (P_j) at rate $f_{I_iP_j}$, with conversion efficiency $e_{I_iP_j}$ of energy into an immune cell with loss rate is m_i. Here, the energy to produce immune cells is assumed never limiting, or that it is solely derived from parasite themselves.

Growth rate of parasite P_j (eq. B5.b): The two competing parasites grow logistically with per capita growth rate r_j and carrying capacity k_j. The parasites are lost due to attack by immune cells ($f_{I_iP_j} I_i$) and die at (shared) background rate m_P.

$$\frac{dI_i}{dt} = (\Sigma e_{I_iP_j} f_{I_iP_j} P_j - m_i)\, I_i \qquad \text{(B5.a)}$$

$$\frac{dP_j}{dt} = \left(r_j(1 - \frac{P_j}{k_j.}) - \Sigma f_{I_iP_j} I_i - m_P\right) P_j \qquad \text{(B5.b)}$$

The Jacobian matrix, feedback loops, and key ratios:

First, the Jacobian matrix for 2P2I at its' feasible interior equilibrium, $\mathbf{J_{2P2I}}$, in order of I_1, I_2, P_1, P_2 is:

$$\mathbf{J_{2P2I}} = \begin{bmatrix} 0 & 0 & J_{I_1P_1} & J_{I_1P_2} \\ 0 & 0 & J_{IP_1} & J_{I_2P_2} \\ J_{P_1I_1} & J_{P_1I_2} & J_{P_1P_1} & 0 \\ J_{P_2I_1} & J_{P_2I_2} & 0 & J_{P_2P_2} \end{bmatrix} = \begin{bmatrix} \mathbf{L} & \mathbf{I} \\ \mathbf{S} & \mathbf{C} \end{bmatrix} \qquad \text{(B6)}$$

with submatrices for limiting niche factors i.e., the immune system here (**L**), impacts on niche (**I**), sensitivity to niche (**S**), and direct competition (**C**).

$$\mathbf{L} = \begin{bmatrix} 0 & 0 \\ 0 & 0 \end{bmatrix}, \mathbf{I} = \begin{bmatrix} J_{I_1P_1} & J_{I_1P_2} \\ J_{I_2P_1} & J_{I_2P_2} \end{bmatrix}, \mathbf{S} = \begin{bmatrix} J_{P_1I_1} & J_{P_1I_2} \\ J_{P_2I_1} & J_{P_2I_2} \end{bmatrix}, \mathbf{C} = \begin{bmatrix} J_{P_1P_1} & 0 \\ 0 & J_{P_2P_2} \end{bmatrix}. \qquad \text{(B7)}$$

As seen above, the stability of the interior equilibrium - determining coinfection vs. priority effects - depends on the sign of level 4 feedback, F_4. Coinfection requires negative level 4 feedback, $F_4 < 0$, at a feasible interior. This level of feedback is the determinant of the Jacobian (F_4 = -det[$\mathbf{J_{2P2I}}$]). It can be written three different ways:

$$F_4 = \underbrace{\underbrace{J_{I_1P_1}J_{P_2I_1}J_{I_2P_2}J_{P_1I_2}}_{P_1 \text{ attacks } P_2 \text{ via } I_1} + \underbrace{J_{P_1I_1}J_{I_1P_2}J_{P_2I_2}J_{I_2P_1}}_{P_1 \text{ attacks } P_2 \text{ via } I_2}}_{\textbf{Inters}\text{–pecific competition}} \underbrace{- \underbrace{J_{I_1P_1}J_{P_1I_1}J_{I_2P_2}J_{P_2I_2}}_{P_1 \text{ attacked by } I_1;\ P_2 \text{ attacked by } I_2} - \underbrace{J_{I_1P_2}J_{P_2I_1}J_{I_2P_1}J_{P_1I_2}}_{P_2 \text{ attacked by } I_1;\ P_1 \text{ attacked by } I_2}}_{\textbf{Intra}\text{–specific competition}} \qquad \text{(B8.a)}$$

$$F_4 = -\det[\mathbf{L\,C - I\,S}] = -\det[\mathbf{I}]\det[\mathbf{S}] = -(J_{I_1P_1}J_{I_2P_2} - J_{I_1P_2}J_{I_2P_1})(J_{P_1I_1}J_{P_2I_2} - J_{P_1I_2}J_{P_2I_1}) \qquad \text{(B8.b)}$$

$$F_4 = -J_{P_1I_1}J_{I_1P_1}J_{P_2I_2}J_{I_2P_2}\, x \underbrace{\left(\frac{J_{P_1I_2}}{J_{P_1I_1}} - \frac{J_{P_2I_2}}{J_{P_2I_1}}\right)}_{sensitivity\ ratio\ (\alpha_1 - \alpha_2)} x \underbrace{\left(\frac{J_{I_2P_1}}{J_{I_1P_1}} - \frac{J_{I_2P_2}}{J_{I_1P_2}}\right)}_{impact\ ratio\ (\varepsilon_1 - \varepsilon_2)} \qquad \text{(B8.c)}$$

In the first way (B8.a), F_4 is written as the sum of two positive (destabilizing) and two negative (stabilizing) loops. The first two positive loops are competitive interactions via the other parasite species (*inter*specific competition), while the last two negative loops are competitive interactions of each species on itself via resource competition (*intra*specific competition). Then, the strength of intra- vs inter-specific

competition governs coexistence vs priority effects. When relative effects of intra- is greater than inter-specific competition then coexistence ensues; vice-versa for priority effects.

In the second way (eq. B8.b), we use a formula for the determinant of block matrices made of the 2x2 squares of the same size (i.e., **L**, **C**, **I**, **S**; eq. B7). Notice here, the logistic growth of the parasite creates a set of parasite self-limitation loop ($J_{P_1P_1}, J_{P_2P_2}$) only, while the previous models (2PIEi) had only the self-limitation via resources but not parasite self-limitation. These self-limitation loops only contribute to the lower level of feedback. However, in all cases the stability of the interior equilibrium depends on the sign of feedback looped through all four interacting species and niche factors (level 4 feedback, F_4; feedback looped through 1-3 interactors all are negative). Therefore, at this level, the lower level (self-limitation) feedback does not contribute to the sign of F_4. Thus, coinfection requires negative feedback, $F_4 < 0$, at a feasible interior. Further simplification of the matrix shows that feedback only depends upon the feedback of the sensitivity-to, -det(**S**), and of the impacts-on submatrix, -det(**I**) since **L** = **0**. Here, the niche (**L**) is the immune system, i.e., if immune cells limited themselves via interference or had some energy limitation, these would not be zero anymore. Feedback at level 4 is negative if both **S** and **I** submatrices produce negative feedback - or if both determinants of **S** and **I** are positive.

Finally, in the third variation (B8.c), that four-species loop product can be reorganized as the *sensitive* (α_j) – *impacts* (ε_j) ratio on their two types of immune cells (arms of immunity). Here, the *sensitivity ratio* is the net effect of immune cell 2 to immune cell 1 on P_j, *i.e.,* increasing either immune cell density has a negative effect on species *j*. Additionally, the *impacts* ratio for each P_j, is the ratio of the effects of P_j on immune cell 2 *vs* 1. Then, the *impacts* ratio is the net effect of density of P_j on growth rate of immune cells, *i.e.,* increasing parasite density has a positive effect on immune cell density. Stability hinges on (a)symmetry of these ratios. Here, coinfection minimally requires each species to trade off their, say, resistance requirements for each immune cell type. If each parasite has larger *impact* on the immune arm to which its fitness is most *sensitive*, it yields a symmetry of *sensitivity-impact* ratios ($\varepsilon_1 > \varepsilon_2$ and $\alpha_1 > \alpha_2$, respectively) enabling coinfection. For priority effects, the competition trade-off did not change ($\alpha_1 > \alpha_2$) but the *impacts* ratios flip ($\varepsilon_1 < \varepsilon_2$). Hence, when each parasite has greatest impact on the niche dimension to which its competitor is most sensitive, net positive feedback and priority effects prevail.

C. Notes on a case study: In synthesizing the literature, we drew on a variety of extant experiments to make connections to the theoretical predictions outlined. Each study includes different treatments and is contextualized in unique ways. In this section, we explain how we replot the data from select studies, both to pay homage to the original work and to clarify the rationale behind our replotting approach.

Replotting of Data from Fellous & Koella (2009): In Fellous & Koella (2009), uninfected larvae of the mosquito *Aedes aegypti* were reared and exposed to two concentrations of the microsporidium *Vavraia culicis* (1000 or 10000) and the protozoan *Ascogregarina culicis* (500 or 50000), with two levels of food (low or high level of fish food). The study primarily focused on the effects of dose and reported interactions with food across various metrics of infection (see Fellous & Koella, 2009 for further details).

Here, we replotted the raw data made available to us, primarily focusing on the impacts of nutrient food supply and only considering treatments exposed to both parasites (excluding single infections). First, across all spore doses, we examined log-transformed parasite load on the individual scale, categorizing the outcomes as follows (Fig. 8C): successful coinfection if both species persisted (orange shaded); P_1 wins (or *Vavraia* wins; blue shaded) if only *Vavraia* persisted but not *Ascogregarina*; P_2 wins (or *Ascogregarina* wins; purple shaded) if only *Ascogregarina* persisted; no infection if neither species was able to persist (yellow). Second, for only those individual hosts that were successfully coinfected (orange shaded), we plotted the change in mean density of parasite load across food levels for the various dose combinations using 95% bootstrapped confidence intervals (R package: boot; Canty & Ripley, 2023). Across all dose combinations, similar trends were observed: P_2 (*Ascogregarina*; purple) increased with more food, while P_1 (*Vavraia*; blue) decreased (Fig. S1). At a minimum, this supports the "equilibrial" view—regardless of dose, the shifting of community structure across nutrient gradients still holds. For detailed effects of dose and its interactions with food on parasite load *see* Fellous & Koella 2009. Finally, for generality, we pooled data across all dose combinations as presented in Fig. 1C, 8B (Case II).

Fig. S1. *Coinfection community structure:* Coinfecting parasites can alter both the relative and absolute abundance of species within hosts as resource supply increases (S). Empirically, shifts in community structure are observed in mosquito host larvae coinfected by microsporidians and protozoan (case II: Fig. 8B; Fellous & Koella, 2009). Only successfully coinfected hosts are shown, with changes in mean parasite density across food levels for various initial dose combinations of *Vavraia* (*Vav*; 1000 or 10000) and *Ascogregarina* (*Asc*; 500 or 5000). Large black dots represent the means, with error bars indicating 95% bootstrapped confidence intervals. Across all dose combinations, similar trends were observed: P_2 (*Ascogregarina*; purple) increased with more food, while P_1 (*Vavraia*; blue) decreased. For generality, data across all dose combinations were pooled, as presented in Fig. 1C and 8B.

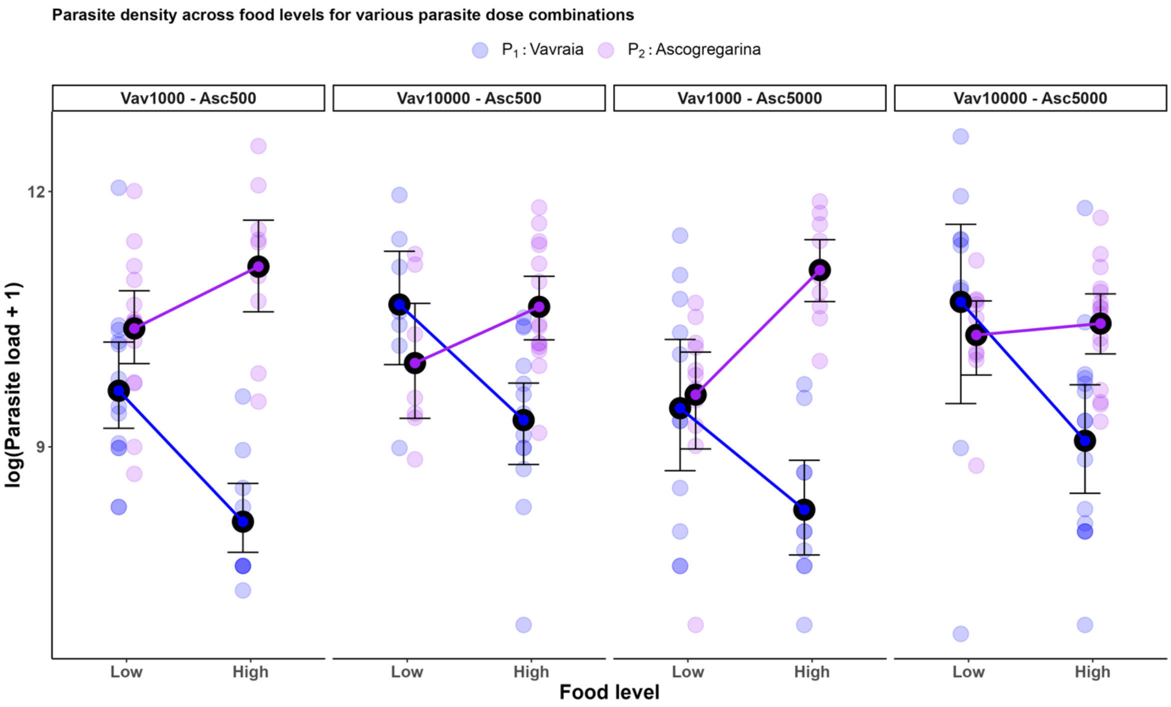

Supplement: Supplementary file 1 — Data S1. [file ELE-28-0-s001.pdf]
